# Unlocking the potential of willow condensed tannins: effects on rumen fermentation, microbiome, and metabolome for sustainable ruminant nutrition

**DOI:** 10.1186/s42523-025-00444-6

**Published:** 2025-07-25

**Authors:** Joshua P. Thompson, Omar Cristobal-Carballo, Tianhai Yan, Katie Lawther, Nicholas J. Dimonaco, Wayne E. Zeller, Zhenbin Zhang, Sharon Huws, Laudina Safo, Andrew D. Southam, Christian Ludwig, Gavin R. Lloyd, Sokratis Stergiadis, Katerina Theodoridou

**Affiliations:** 1 Institute for Global Food Security, Queen’s University Belfast, Northern Ireland, Belfast, UK; 2 School of Biological Sciences, Queen’s University Belfast, Northern Ireland, Belfast, UK; 3 School of Agriculture, Policy and Development, University of Reading, Reading, Berkshire UK; 4 Sustainable Livestock Systems Branch, Agri-Food and Biosciences Institute, Hillsborough, UK; 5 USDA-ARS, US Dairy Forage Research Center, Madison, WI USA; 6 Phenome Centre Birmingham, School of Biosciences, University of Birmingham, Edgbaston, Birmingham, B15 2TT UK; 7 Department of Metabolism and Systems Sciences (MSS), College of Medicine and Health, University of Birmingham, West Midlands, UK

**Keywords:** Methane, Willow, Fermentation, Tannins, Ruminant, Ammonia, Metabolomics, Microbiome

## Abstract

**Background:**

Sustainable livestock production is essential for meeting the growing global protein demand while minimising environmental impacts. Exploring alternative forages that enhance nutrient utilisation and reduce reliance on imported feeds is a potential strategy. Condensed tannins (CTs) can bind to proteins in the rumen, protecting them from ruminal degradation resulting in decreased ammoniacal N and enhanced nitrogen uptake in the hindgut. This pioneering research is the first to explore the potential of willow (*Salix*) as an alternative feed for ruminant nutrition. The study involved feeding ewe hoggets a control grass silage (SIL) or a SIL mix containing a 20% dry matter (DM) dietary inclusion of leaves from two willow varieties to investigate the impact the willow CTs have on rumen fermentation, microbial populations, and metabolomic profiles. Willow treatments: Beagle (BG) and Terra Nova (TN) had an overall CT inclusion (CTI) of 1.1 and 0.1% DM with the control diet containing no CTs in a three-treatment x three-period Latin square design.

**Results:**

Although total dry matter and fibre intake were higher in BG and TN, there was no significant difference in ruminal CH_4_ production between the treatments. However, fermentation was affected, with BG and TN showing lower acetate production and reduced total volatile fatty acid production compared to SIL. CTs may have impaired fibre digestion, as SIL had higher *Fibrobacter* abundance than BG. Heatmap visualisation indicated higher carbohydrate metabolite concentrations in SIL, with reduced metabolism observed in TN and BG. Ruminal ammonia did not differ significantly among treatments, despite higher nitrogen intake in BG and TN treatments. Proteolytic bacteria levels were similar across treatments, but TN and BG had higher ruminal metabolites associated with protein metabolism upon visualisation through heatmap analysis. TN showed higher abundance of *Prevotella* and *Fibrobacter* than BG, which had 10 times higher CT content and a greater prodelphinidin proportion.

**Conclusion:**

Feeding CT-containing willow enhanced feed intake, altered rumen microbiome composition and suggested visual changes in the analysis of protein metabolism, offering potential benefits for animal performance. While a reduction in CH_4_ was not observed, this study highlights the potential of willow to alter ruminant nutrition while supporting sustainable agricultural practices.

**Supplementary Information:**

The online version contains supplementary material available at 10.1186/s42523-025-00444-6.

## Background

Ruminant-derived products contribute a nutrient-dense food source that plays a vital role in human health [1]. It is the rumen microbiome that enables ruminants to convert plant biomass into digestible nutrients, which are subsequently available for human nutrition [2]. Nevertheless, ruminant systems are under pressure to address efficiency issues associated with their production. Rumen fermentation and its microbiome play a key role in tackling these efficiency challenges. Dietary supplementation with bioactive compounds has been shown to effect rumen fermentation pathways causing changes in volatile fatty acid production alongside changes in rumen microbiome composition.

If these changes lead to an increase in propionate production alongside a decline in acetate, a reductions in ruminal methane production could be observed [3]. Furthermore, fermentation pathways using N can be altered by supplementation of bioactive compounds that can inhibit hyper ammonia producing bacteria. This can limit ruminal nitrogen degradation increasing the amount of undegraded nitrogen reaching the abomasum for maintenance and growth requirements [3].

Dietary supplementation with condensed tannins (CTs) presents promising opportunities for the ruminant industry. CTs are well known for their ability to bind to proteins in anaerobic conditions [4]. In the rumen, a neutral pH favours the formation of CT-protein complexes reducing ruminal protein degradation and production of ammonia-N (NH_3_-N) [5]. Consequently, there is a greater flow of non NH_3_-N to the abomasum where a more acidic pH causes a dissociation of the CT-protein complex, releasing protein to be available for duodenum uptake and metabolism [5]. Additionally, CTs can bind to fibre reducing fibre digestion and volatile fatty acid production [6]. This results in less hydrogen being produced, indirectly leading to a reduction in ruminal CH4 production [10]. However, CT-bound fibre can reduce the availability of digestible energy, which may negatively impact growth [11].

In addition to binding to protein and fibre in the rumen, CTs can have different modes of action. It has been proposed that CTs can prevent or interfere with the attachment of rumen microbes to feed particles reducing fermentation [5]. Secondly, CTs can reduce the degradation of protein and fibre through enzyme inhibition, with studies reporting CTs can reduce the activity of hemicellulases, endoglucanases and proteolytic enzymes [7, 8]. Thirdly, CTs can have an anti-microbial effect in the rumen. One study shows CT supplementation with goats resulted in a decline of phylum *Firmicutes*, whereas phylum *Bacteroidetes* increased in the goat rumen [9]. In addition, several studies have shown CT supplementation to effect concentrations of *Ruminococcus flavefaciens*, *Fibrobacter succinogenes* and *Butyrivibrio fibrisolvens* [7, 8, 10, 11]. Furthermore, CTs can have an anti-microbial effect on methanogens with supplementation in sheep shown to lower methanogen concentrations in the rumen despite no influence on the overall community structure of the methanogen [9, 10, 12].

Another way to assess the impact of CTs on rumen fermentation and microbiome is through the characterisation and quantification of ruminal metabolites. If the rumen microbiome is affected by CTs, metabolomics will reveal changes in the pathways of nutrient degradation, providing insight into the functionality of the altered microbiome [13].

However, the effect of CTs on fermentation and the microbiome varies widely between sources and the structural arrangement of CTs. Larger molecular size, mean degree of polymerisation (mDP) and prodelphinidin (PD) content have been correlated with greater impacts on rumen fermentation and CH_4_ reduction [14, 15]. This is due to the larger size and the greater the number of repeating flavan-3-ol subunits increasing the binding capacity of the CT. Also, a greater proportion of PD flavan-3-ol subunits compared to procyanidins (PC) flavan-3-ol subunits in the CTs again enhance the binding capacity as the PD flavan-3-ol subunit’s structure has a greater number of sites for hydrogen bonding interactions, enhancing their ability to bond to fibre and protein [15].

Willow (*Salix* sp.) fodder is currently used in biofuel production, however leaves and branches up to 18 mm diameter are not used from these harvests, and constitute a considerable waste stream [16]. This willow tree fodder stream has a high protein content which is well above that required for livestock maintenance and contains CTs. Therefore, supplementing ruminants with willow fodder could have potential to directly and indirectly change fermentation pathways of ruminants improving efficiency problems associated with their production. This pioneering research is the first to explore the potential of willow (*Salix*) as an alternative feed for ruminant nutrition and to investigate the impact of its CTs in the leaves of two different varieties: Beagle (BG) and Terra Nova (TN) on rumen fermentation, microbiome populations and metabolome profiles of growing female sheep aged between one and two years (ewe hoggets).

## Results

### Condensed tannin (CT) content, structure and inclusion

Freezing as a preservation method of CT-containing willow forages had significant effects on both CT content and structure. Upon freezing, the bound CT content of both BG and TN increased by 82% (*P* < 0.001; Table 1) and 70% (*P* < 0.05, Table 1), respectively. Alternatively, both unbound and total CT content of BG decreased by 47% (*P* < 0.01; Table 1) and 33% (*P* < 0.01; Table 1), respectively upon freezing. The decrease was even greater for TN with unbound and total CT content decreasing by 94% (*P* < 0.05; Table 1) and 78% (*P* < 0.05; Table 1), respectively. The effect of freezing on CT structure had differing affects between BG and TN. For BG, mDP was decreased by 16% (*P* < 0.05; Table 1) while the proportion of PC increased as proportion of PD decreased (*P* < 0.01; Table 1). For TN, no significant differences were observed in mDP between fresh and frozen – though mDP could not be determined on two out of the three samples due to interference form impurity signals. However, similar to both willow varieties was the significant increase in the %A-type linkages after freezing with no A-type linkages found in the fresh forms of CT from BG and TN. The greatest increase in the %A-type linkages was TN with 5.97% vs 0.67% for BG.

**Table 1:** The effect of preservation (fresh vs frozen) on condensed tannin content and structure of each willow variety used in the study

	Willow variety							
	BG				TN			
	Preservation				Preservation			
	Fresh	Frozen	s.e.m	P-value	Fresh	Frozen	s.e.m	*P*-value
*Content (%DM)*								
Bound	1.56	2.84	0.222	***	0.740	1.26	0.114	*
Unbound	12.1	6.39	1.09	**	6.81	0.408	1.05	*
Total	13.6	9.05	0.896	**	7.55	1.65	0.968	*
*Structure*								
mDP	8.75	7.31	0.285	*	10.4	14.0	1.88	0.374
%PC	32.8	37.3	0.434	**	95.3	94.6	1.24	0.402
%PD	67.2	62.7	0.434	**	4.72	5.42	1.24	0.402
%cis	21.5	20.0	0.760	0.140	65.0	64.0	1.08	0.694
%trans	78.5	80.0	0.760	0.140	35.0	36.0	1.08	0.694
%A-type	0.00	0.670	0.144	*	0.00	5.97	0.829	*

SIL, silage; BG, Beagle willow variety; TN, Terra Nova willow variety; mDP, mean degree of polymerisation; PC, procyanidin, PD, prodelphinidin; %A-type, the % of A-type linkages intramolecularly within CTs; The symbols ‘*’, ‘**’,’***’, denote significance *P* < 0.05, 0.01 and 0.001 respectively

**Fig. 1:**
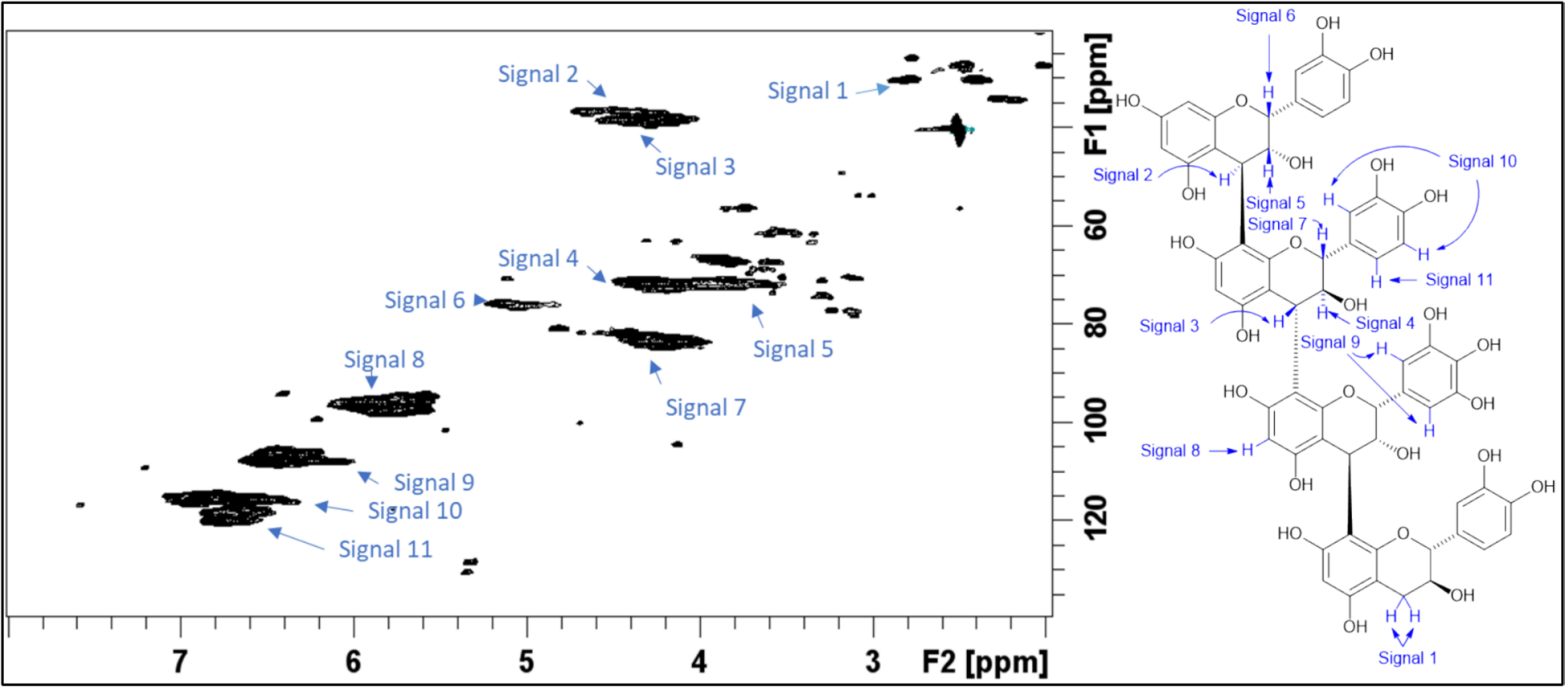
^1^H-^13^C HSQC NMR spectrum of the Salix Beagle CT reference standard with signals identified as listed in the accompanying condensed tannin structure

**Fig. 2:**
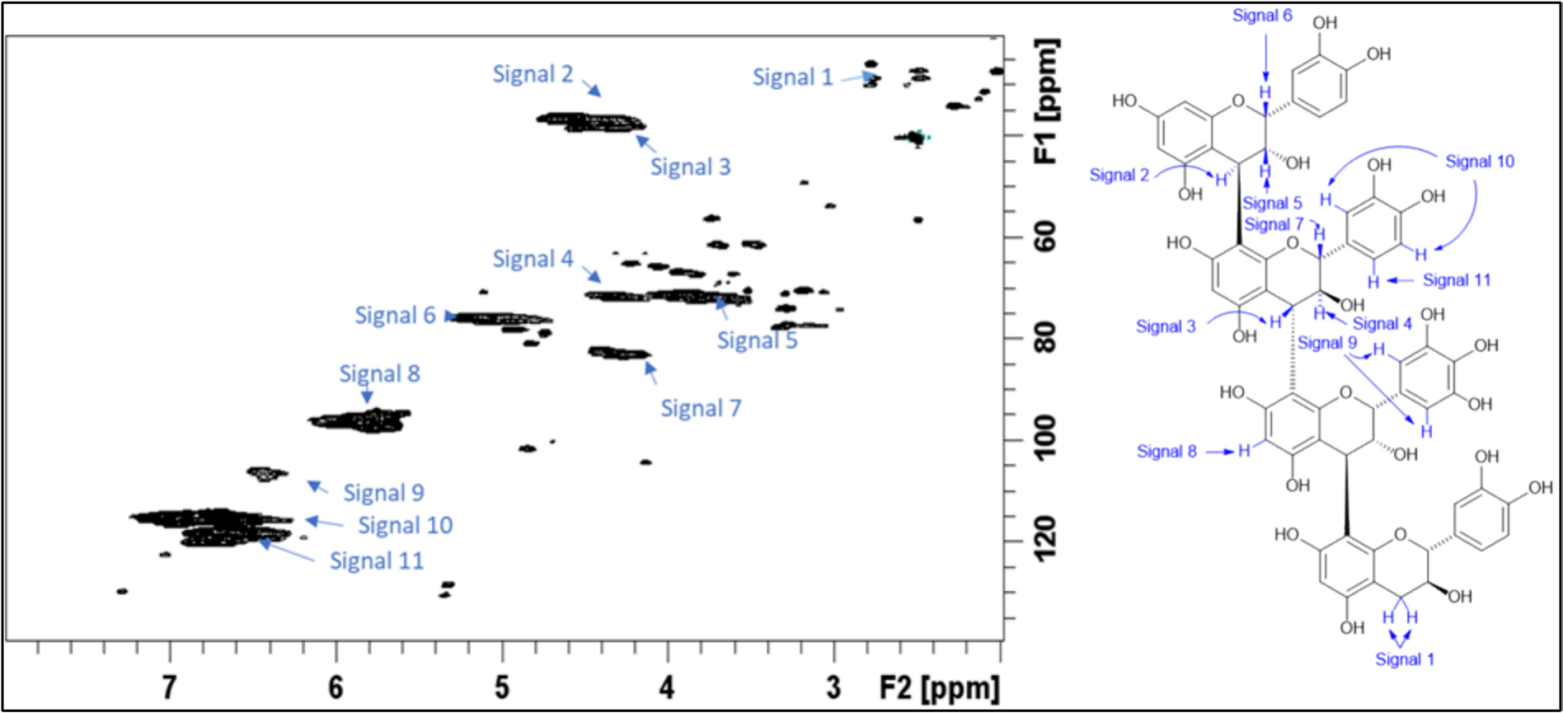
^1^H-^13^C HSQC NMR spectrum of the Salix Terra Nova CT reference standard with signals identified as listed in the accompanying condensed structure

The two-dimensional (2D) ^1^H-^13^C Heteronuclear Single Quantum Coherence (HSQC) Nuclear Magnetic Resonance (NMR) spectra of the purified CTs (reference standards) from fresh samples *Salix* Beagle and *Salix* Terra Nova are displayed in Figs. 1 and 2, respectively, with Table 2 displaying the differences in CT content and structural determination data between BG and TN obtained from the 2D NMR spectra. In terms of CT content BG had significantly greater bound, unbound and total CT content relative to TN which consequently will affect CT intake (CTI) between the two treatments. Moreover, contrasting features are evident between CT structural arrangements. First, BG CT had an average *cis/trans* (epicatechin + epigallocatechin to catechin + gallocatechin) ratio of approximately 1:4, whereas the average *cis/trans* ratio of TN CT were about 2:1 (P < 0.001; Table 2). Second, average PC/PD (catechin + epicatechin to gallocatechin + epigallocatechin) ratio for BG are approximately 1:2 whereas the average PC/PD ratio for TN is approximately 95:5 (*P* < 0.001; Table 2). Thirdly, the proportion of A-type linkages in the frozen samples were 9 times greater in TN compared to BG (*P* < 0.001; Table 2).

**Table 2:** Condensed tannin content and structure of the three different treatments used in this study

	Willow Variety				
	SIL	BG	TN	s.e.m	*P*-value
Bound	Nd	2.84	1.26	0.248	***
Unbound	Nd	6.38	0.41	0.936	**
Total	Nd	9.05	1.65	1.16	**
*Structure*					
mDP	Nd	7.31	4.66	1.19	0.224
%PC	Nd	37.3	94.6	7.00	***
%PD	Nd	62.7	5.42	7.00	***
%*cis*	Nd	20.0	64.0	5.38	***
%*trans*	Nd	80.0	36.0	5.38	***
%A-type	Nd	0.670	5.97	0.673	***

SIL, silage; BG, Beagle willow variety; TN, Terra; Nova willow variety; mDP, mean degree of polymerisation; PC, procyanidin; PD, prodelphinidin; %A-type, the % of A-type linkages intramolecularly within CTs; Nd, none detected; The symbols ‘*’, ‘**’,’***’, denote significance *P* < 0.05, 0.01 and 0.001 respectively

In this study, we used the unbound CT determination for forage (Supplementary Table S1) and formulated diet (Supplementary Table S2) CT content. Unbound CTs are free to react and influence feed particles or the rumen microbiome. For BG and TN, the formulated diets consisted of 12.82 and 0.78 g CT/kg DM while no CT were detected in SIL. Due to differences in CT content, CTI was significantly affected by feed treatment (*P* < 0.001; Table 3). Ewe hoggets fed BG had a CTI of 17.0 g/d compared to 1.1 g/d for TN. Overall CT inclusion (%TDMI) was 1.1 for BG and 0.1 for TN (Table 3).

**Table 3:** Animal and nutrient intakes of ewe hoggets for each treatment

	Feed Treatment				
	SIL	BG	TN	s.e.m	*P*-value
*Animal intake*					
FDMI (g/d)	1070.8^a^	1327.3^b^	1371.7^b^	24.3	***
CDMI (g/d)	160.0	160.0	160.0	–	–
TDMI (g/d)	1230.8^a^	1487.3^b^	1531.7^b^	24.3	***
OMI (g/d)	1119.1^a^	1357.4^b^	1395.2^b^	22.3	***
WI (%DM)	0.0^a^	20.1^b^	19.1^c^	0.6	***
CTI (% Total DMI)	0.0^a^	1.1^b^	0.1^c^	0.03	***
*Nutritive intakes*					
GEI (MJ/d)	23.7^a^	28.9^b^	29.8^b^	0.5	***
MEI (MJ/d)	13.6^a^	16.3^b^	17.3^b^	0.3	***
ADFI (g/d)	396.9^a^	474.4^b^	513.1^b^	8.6	***
NDFI (g/d)	621.5^a^	718.8^b^	776.0^b^	12.9	***
NI (g/d)	31.0^a^	40.0^b^	41.8^b^	0.7	***
CTIntake(g/d)	0.0^a^	17.0^b^	1.1^c^	0.5	***

SIL, Silage control; BG, Salix. Beagle; TN, Salix Terra Nova; FDMI, forage dry matter intake; CDMI, concentrate dry matter intake; TDMI, total dry matter intake; OMI, organic matter Intake; WI, willow inclusion; CTI, condensed tannin inclusion DM,dry matter; CT, condensed tannin; GEI, gross energy intake; MEI, metabolisable energy intake; ADFI, acid detergent fibre intake; NDFI, neutral detergent fibre intake; NI, nitrogen intake; SI, starch intake; CTIntake, condensed tannin intake; The symbols ‘*’, ‘**’,’***’, denote significance *P* < 0.05, 0.01 and 0.001 respectively

### 2.2 Animal nutritive intakes

Forage DMI (FDMI; g/d) was 24% and 28% greater for BG and TN (*P* < 0.001; Table 3) relative to SIL with no differences (*P* = 0.43) occurring between BG and TN. Accordingly, total DMI (TDMI; g/d) followed the same trend with treatment as did OMI (*P* < 0.001; Table 3). Willow inclusion (WI; %DM) in the diet relative to the control (SIL; 0% WI) was 20.1% and 19.1% for BG and TN treatments, respectively (Table 3). Nutritive intakes were significantly different by treatment with gross energy intake (GEI; MJ/d) 22% and 25% greater for BG and TN relative to SIL (*P* < 0.001; Table 3) with no differences between BG and TN (*P* = 0.43). Likewise, metabolizable energy intake (MEI; MJ/d) were 20% and 27% greater for BG and TN, respectively, compared to SIL (*P* < 0.001, Table 3) with no differences between BG and TN (*P* = 0.096). Regarding fibre, acid detergent fibre intake (ADFI; g/d) was 20% and 29% greater for BG and TN, respectively (*P* < 0.001; Table 3), relative to SIL. Neutral detergent fibre intake (NDFI; g/d) was significant with a 16% and 25% greater intake for BG (*P* < 0.01) and TN (*P* < 0.001; Table 3), respectively, relative to SIL. Differences in fibre intake between BG and TN were non-significant (*P* > 0.05; Table 3). Regarding nitrogen intake (NI), BG and TN had a 29% and 35%, respectively, higher intake relative to SIL (*P* < 0.001; Table 3).

### Impact of treatment nutritive intakes on rumen fermentation

**Fig. 3:**
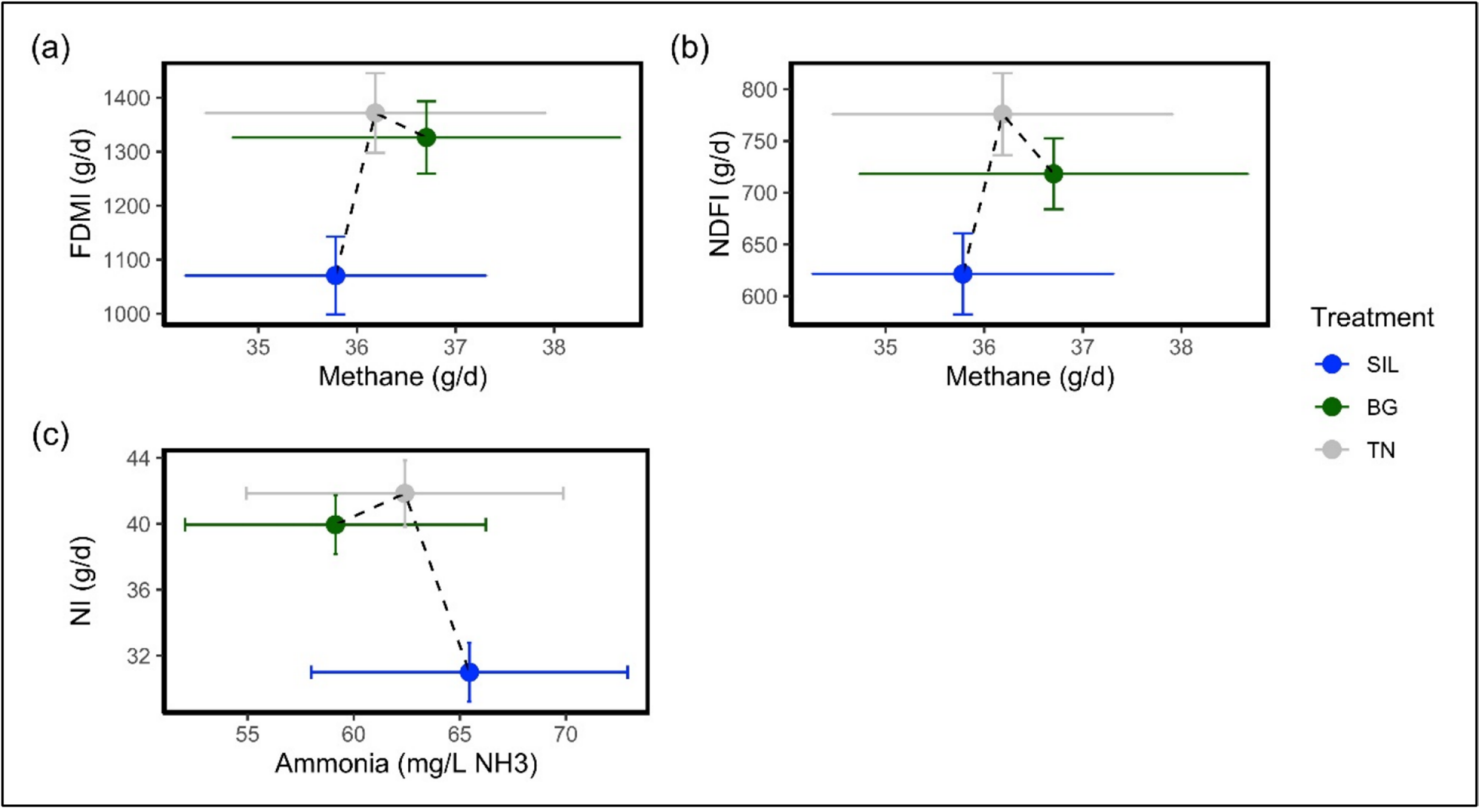
The relationship plot between Nutritional intakes and Fermentation averages of ewe hoggets fed different feed treatments. **a** Daily FDMI (forage dry matter intake) relationship to methane production. **b** Daily NDFI (neutral detergent fibre intake) relationship with methane production. **c** Daily NI (nitrogen intake) relationship with ruminal ammonia concentration. All plots are the averages off all ewe hoggets by feed treatment and include corresponding error bars for each parameter

Ruminal ammonia (NH_3_; mg/L NH_3_) concentration was non-significant between treatments (*P* = 0.678; Table 4) despite a numerically lower NH_3_ concentration of BG (10% reduction; *P* = 0.77; Table 4) and TN (5% reduction; *P* = 0.77; Table 4) relative to SIL. The relationship between nutritive intakes and rumen fermentation parameters are shown in Fig. 3. Despite BG and TN having significantly higher NI, the average NH_3_ production was lower for both the CT containing diets: BG and TN (Fig. 3c). Gas quantities produced from fermentation (CH_4_ and H_2_) were non-significant between all treatments (*P* > 0.05; Table 4). Similarly, energy required for CH_4_ production (CH_4_-E; MJ/d) and the proportion of CH_4_-E of total GEI were non-significant between all treatments (*P* > 0.05; Table 4). Methane emissions per unit of LW, DMI, OMI were non-significant between treatments (Table 4). Nevertheless, Figs. 3a-b show that despite BG and TN having a significantly greater FDMI and NDFI relative to SIL, their CH_4_ production is not significantly higher for the corresponding increased FDMI and NDFI.

**Table 4:** Ruminal fermentation of ewe hoggets feed different feed treatments

	Treatment				
	SIL	BG	TN	s.e.m	*P*-value
Live weight (kg)	65.9	66.5	66.3	1.03	0.972
*Gaseous exchange*					
CO_2_ (g/d)	1330	1360	1340	21.8	0.831
O_2_ (g/d)	1010	1020	1020	16.0	0.929
CH_4_ (g/d)	35.8	36.7	36.2	0.985	0.933
H_2_ (g/d)	0.0509	0.0459	0.0467	0.00291	0.898
RQ	0.962	0.969	0.953	0.00803	0.548
HP (MJ/d)	14.8	15.0	15.0	0.231	0.926
Rumen					
pH	7.14	7.39	7.34	0.0633	*
NH_3_ (mg/L NH_3_)	65.5	59.1	62.4	4.14	0.678
*Methane*					
CH_4_-E (MJ/d)	1.98	2.03	2.00	0.0544	0.933
CH_4_-E/GEI (%)	8.65	7.11	6.87	0.313	0.0950
CH_4_-E/MEI (%)	15.0	12.6	11.8	0.532	0.0660
CH_4_/LW (g/kg)	0.544	0.554	0.549	0.0146	0.970
CH_4_/DMI (g/g)	0.0301	0.0250	0.0242	0.00108	0.101
CH_4_/OMI (g/g)	0.0331	0.0274	0.0265	0.00119	0.103
*Volatile Fatty Acids (mmol/L)*					
Ethanol	0.0242	0.0208	0.0217	0.00120	0.781
Acetic acid	2.20	1.82	1.75	0.141	*
Propionic acid	0.583	0.482	0.472	0.0378	0.0782
i-Butyric acid	0.0358	0.0383	0.0417	0.00429	0.820
n-Butyric acid	0.303	0.300	0.277	0.0193	0.577
i-Valeric acid	0.0450	0.0525	0.0617	0.00486	0.201
n-Valeric acid	0.0242	0.0200	0.0242	0.00260	0.747
A:P	3.82	3.77	3.82	0.101	0.955
Total VFA	3.22	2.73	2.64	0.200	0.0614

RQ, respiratory quotient; HP, heat production; NH_3_, ammonia; CH_4_-E, methane energy; GEI, gross energy intake; MEI, Metabolizable energy intake; LW, live weight; DMI, dry matter Intake; OMI, organic matter intake; A:P, acetic: propionic ratio; VFA, volatile fatty acid. The symbol ‘*’ denote significance *P* < 0.05

**Fig. 4:**
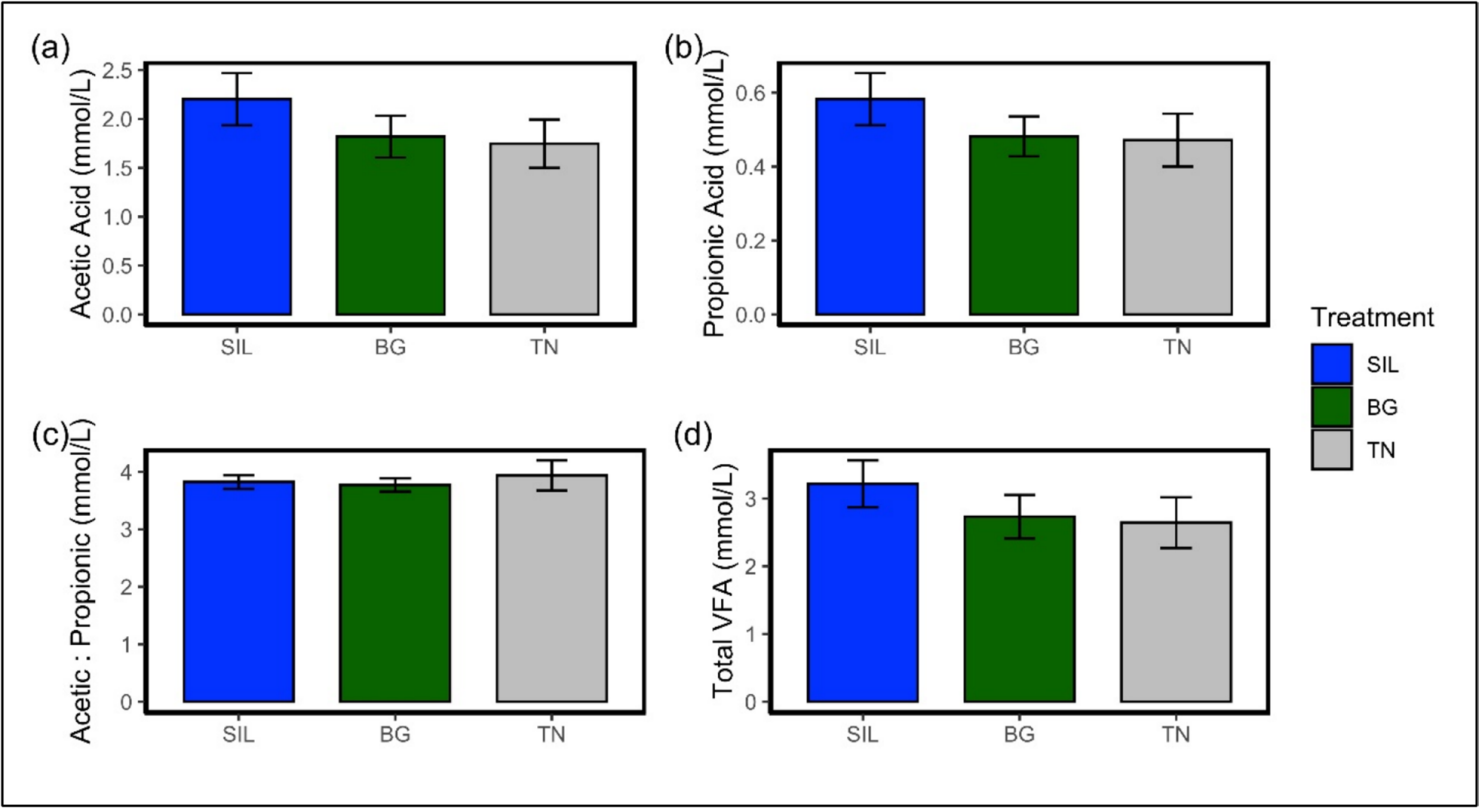
Bar plot of ruminal volatile fatty acid (VFA) production averages of ewe hoggets fed different feed treatments. **a** Average Acetic acid production in the rumen. **b** Average Propionic acid production in the rumen. **c** Average ratio of Acetic to Propionic acid production in the rumen. **d** Average total volatile fatty acid (VFA) production in the rumen. All bar plots contain corresponding error bars for each treatment

Figure 4 shows a comparison of volatile fatty acid (VFA) production in the rumen between treatments. Acetic acid (Fig. 4a) was different by treatment (*P* < 0.05) however no significance was observed between treatment interactions despite BG and TN having a 17% and 21% reduction relative to SIL, respectively. Propionic acid production (Fig. 4b) was non-significant between treatments (*P* = 0.078) despite BG and TN having a numerically reduced concentration of 17% and 19% relative to SIL, respectively. The ratio of acetic acid to propionic acid (Fig. 4c; *P* = 0.955) was non-significant between treatments. Overall, total VFA (tVFA) production was non-significant between treatments (Fig. 4d; *P* = 0.0614). However, there was a tendency for tVFA production to decrease for BG and TN relative to SIL.

### 2.4 CT containing diets affect rumen microbiome composition

A cumulative 1,221,919 circular consensus sequencing (CCS) reads were obtained from PacBio 16S RNA sequencing. The median HiFi read length average across sequencing runs was 1,498 bp, with the median HiFi read quality at Q30 for each sample. After denoising with QIIME2 (v2023.7), 183,527 reads were retained, with 183,465 assigned to amplicon sequence variants (ASVs). Median read counts over the whole trial per animal taxonomically classified were recorded as 3441 for SIL, 3510 for BG and 3145 for TN (Supplementary Figure S1). In terms of taxonomic assignment, over 98.53% distinct ASVs were classified within the Bacteria domain, while 63.45% and 54.83% of ASVs were classified at the genus and species level, respectively.

**Fig. 5:**
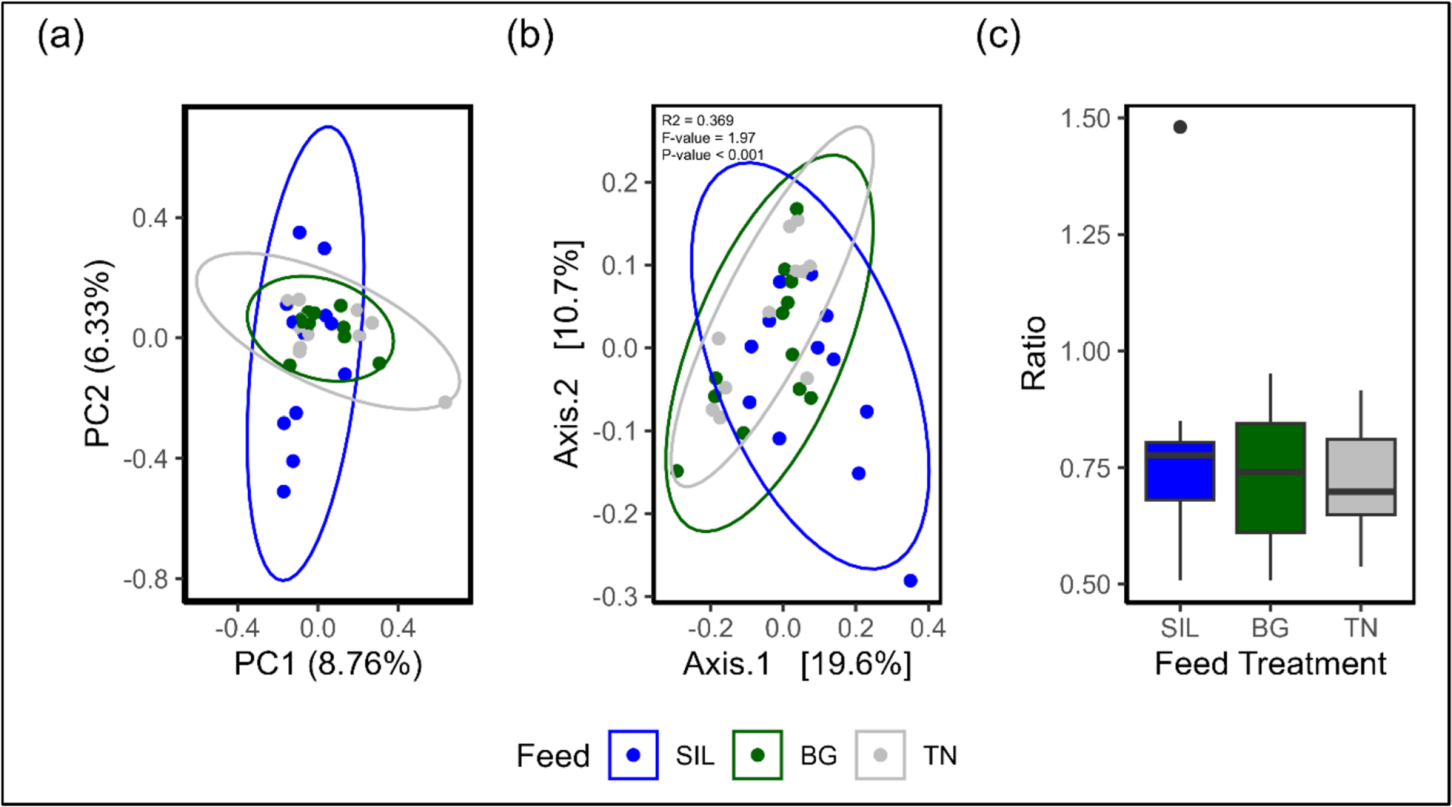
Multivariate analysis, Beta Diversity and phyla composition between feed treatments. **a** PCA plot of ASV read counts after normalization with total-sum scaling transformation. PC1 explains 25.4% of the total variance, while PC2 accounts for an additional 8.75% of the variance. **b** The PCoA plot using the Bray–Curtis distance matrix of ASV read counts after normalization with total-sum scaling transformation. PCoA1 explains 19.6% of the total variance, while PCoA2 accounts for 10.7% of the variance. **c** Ratio of Firmicutes to Bacteroidota relative abundance counts across each feed treatment

PCA of normalised ASV read counts demonstrated BG and TN feed treatments had similar variation with greater variation in the SIL feed treatment (Fig. 5a). Principal components 1 and 2 accounted for 8.76% and 6.33% of the variance, respectively, among rumen communities. Among all analysed feed treatments, BG (137) and TN (136) communities had numerically higher Chao1 (Fig. 5a) richness compared to SIL (125) (*P* = 0.520; Supplementary Table S3), whilst no significant differences were observed for Pielou’s (Fig. 5b; *P* = 0.724) and Inverse Simpson (Fig. 5c; *P* = 0.361) indices (Supplementary Table S3). Beta diversity was visualised using a PCoA plot of normalised ASV counts with Axis 1 and Axis 2 accounting for 19.6% and 10.7% of the variation (Fig. 5b). Feed treatment clusters did overlap, however SIL showed the greatest variation compared to the other treatments. Permanova analysis of beta diversity (Fig. 5b) identified significant differences in the microbial community of different feed treatments (*P* < 0.001).

**Fig. 6:**
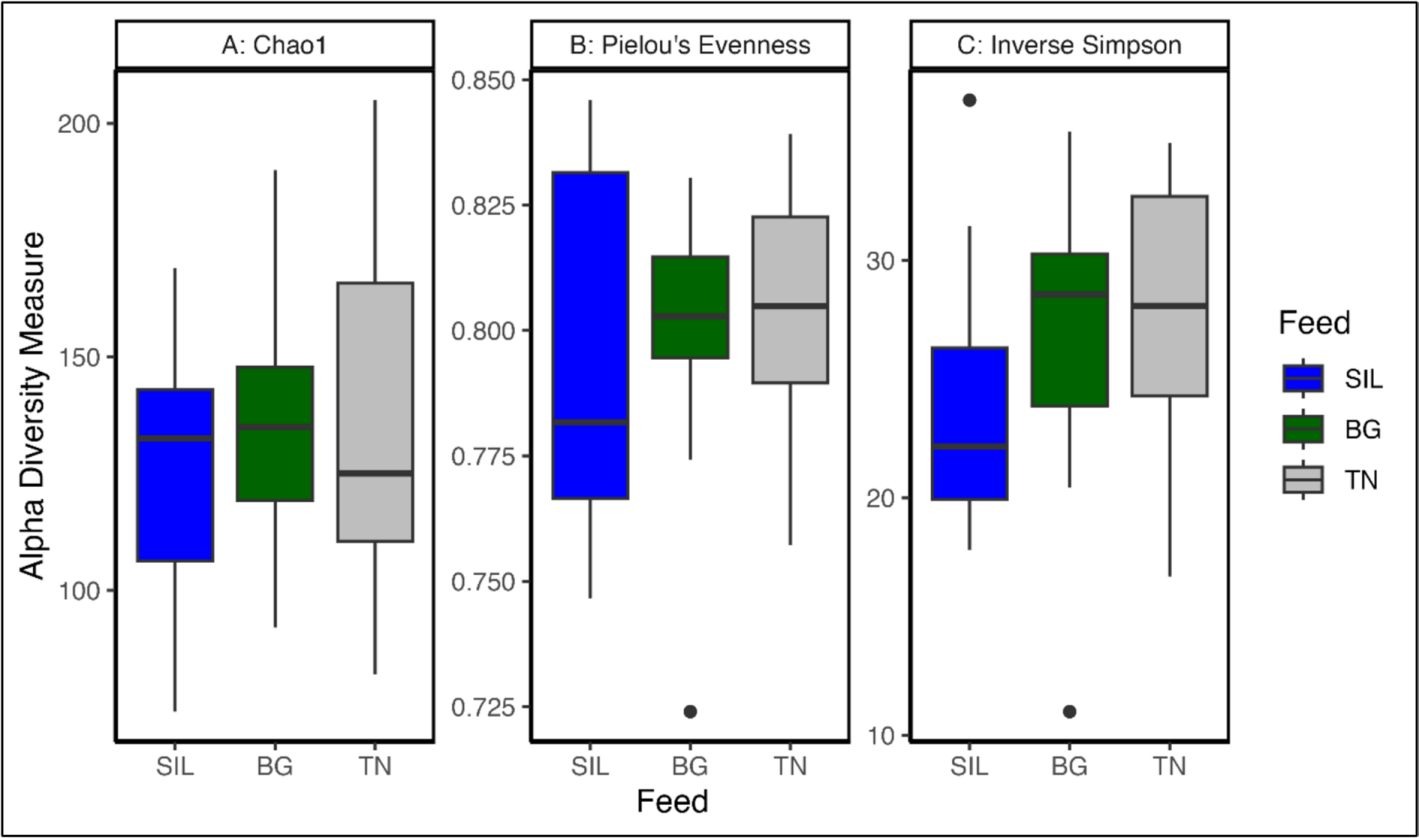
Alpha diversity indices of ASV read counts at genus level. The indices **A** Chao1 Richness, **B** Pielou’s Evenness, and **C** Inverse Simpson Diversity were derived from raw, untransformed ASV read counts. The influence of Feed Treatment was analysed using ANOVA, with significant results presented in Supplementary Table S3

The ratio of phyla Firmicutes to Bacteroidota (Fig. 5c) showed no differences according to feed treatment (P = 0.895; Supplementary Table S4, row 28). Univariate analysis of phyla relative abundance ASV counts revealed Actinobacteriota (*P* < 0.001; Supplementary Table S4, row 10) were significantly different by feed treatment with abundance significantly higher for the CT feed treatments: BG (*P* < 0.05) and TN (*P* < 0.01) relative to SIL. Across all feed treatments, seven phyla accounted for approximately 90% of ASV read count relative abundance. These phyla in descending order, include: Bacteroidata, Firmicutes A, Verrucomicrobiota, Planctomycetota, Firmicutes D, Patescibacteria and Firmicutes C (Supplementary Figure S2 and Table S4). Across all analysed samples, 131 bacterial and one archaeal family (Methanobacteriaceae) were identified. The most abundant families across all feed treatments represented over 51% of the taxonomically classified ASV, including *Bacteroidaceae*, *Lachnospiraceae, Thermoguttaceae, UBA932, UBA660 and UBA1067* (Supplementary Figure S3 and Table S5) (Fig. 6).

**Fig. 7:**
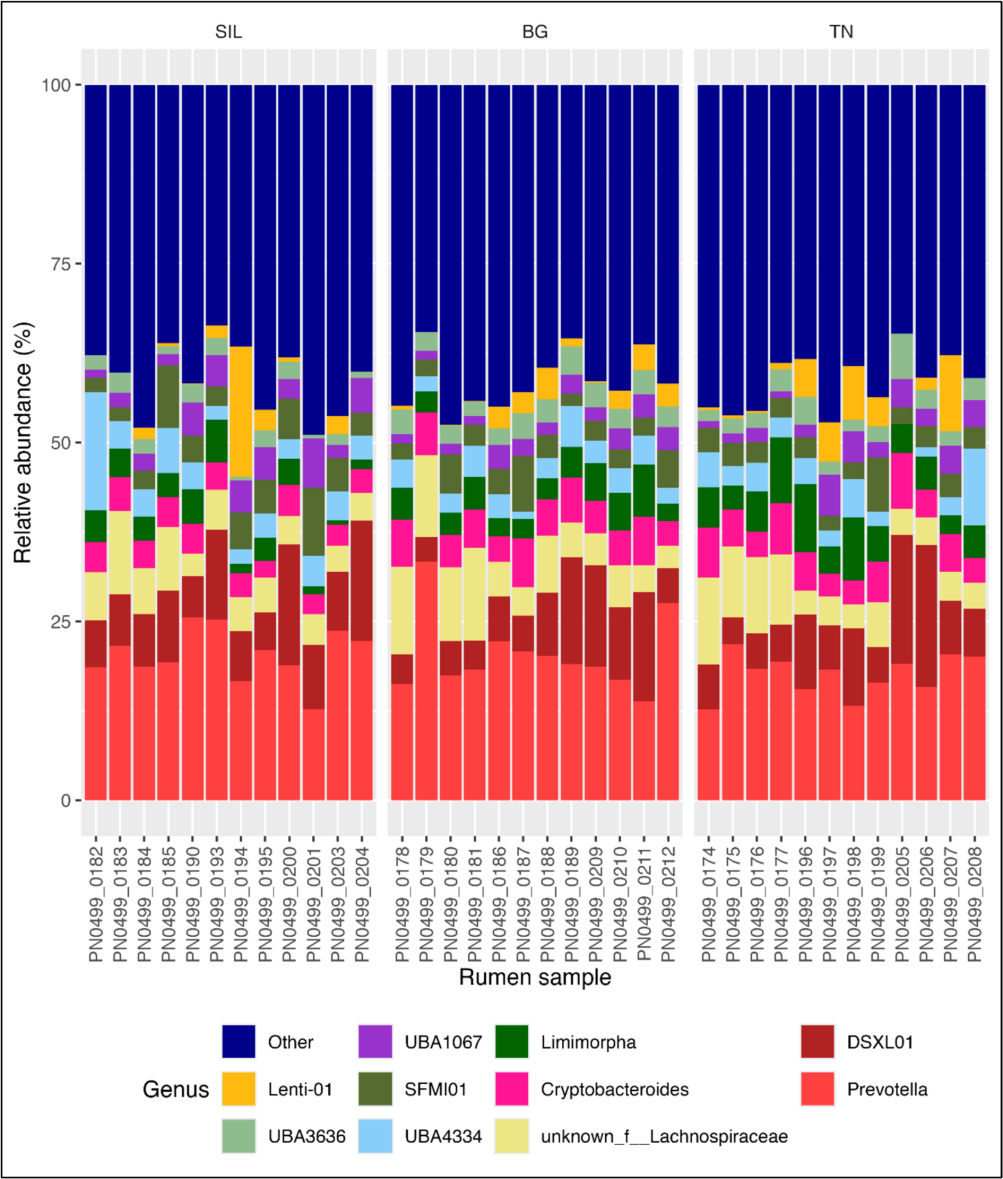
Top 10 most abundant genera of relative abundance in rumen samples. Stacked bar charts illustrate the average relative abundances in percentage, which were calculated by total sum scaling of read counts classified at genus level. The top ten most abundant taxonomic groups are visualised with all other genus aggregated and labelled as ‘Other’. Samples were indicated on the X-axis while relative abundance (%) indicated on the y-axis. Each colour in the chart corresponds to a different predominant genus, as indicated in the legend. Each panel corresponds to a Feed treatment

**Fig. 8:**
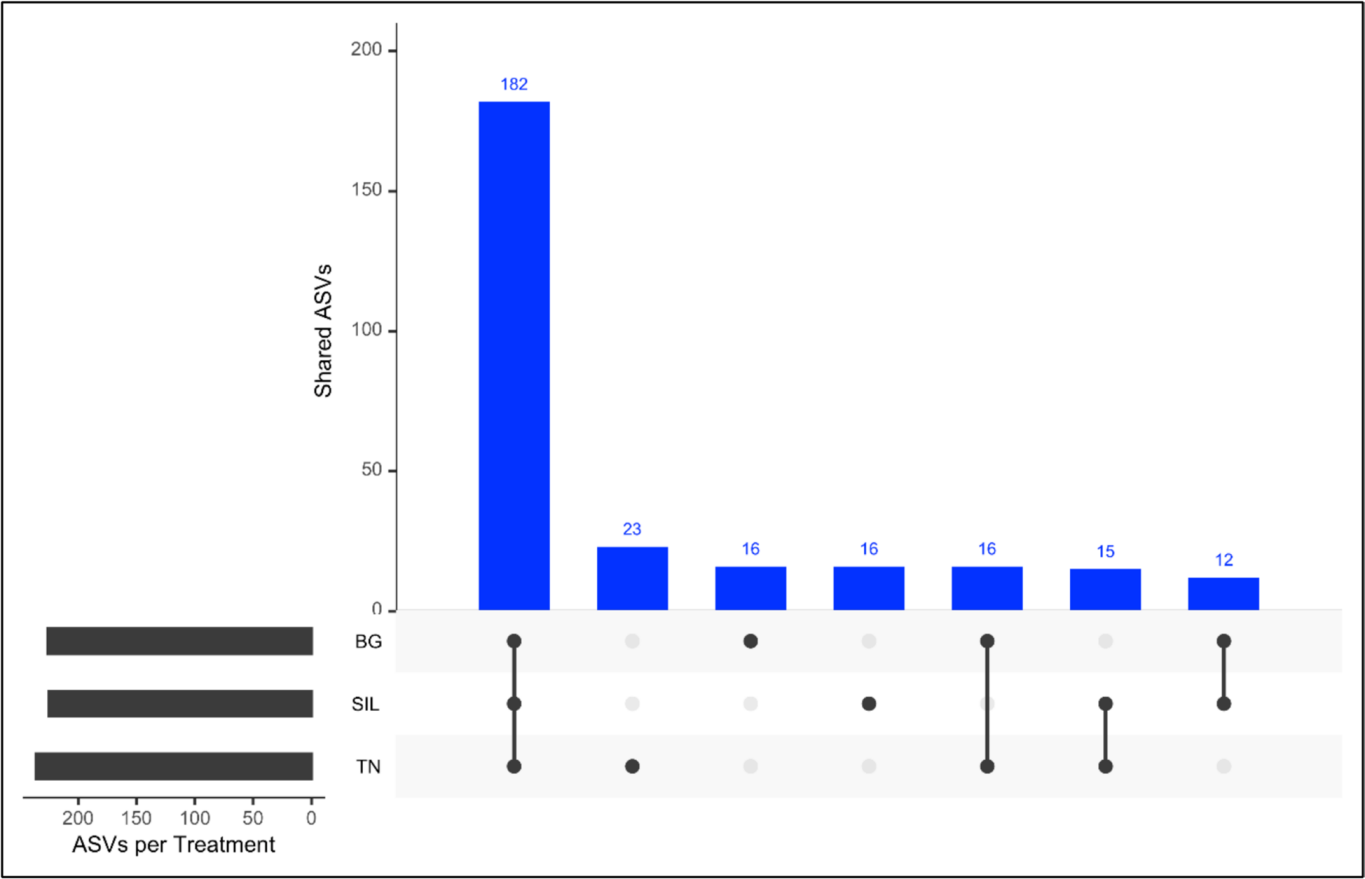
Distribution of genera occurrence across sample types. A total of 182 genera were identified within rumen samples. The upset graph illustrates occurrences of ASV across samples. The number at the top of each bar indicates the quantity of genera observed in the corresponding combination of Feed Treatments

At the genus level, Fig. 7 shows the relative abundance of the top ten most abundant genera for all rumen samples across different feed treatments. Across all rumen communities, *Prevotella* was most abundant across all feed treatments, accounting for 20.4%, 20.4% and 17.6% in SIL, BG and TN, respectively (Table 5). This was followed by *DSXL01*, *unknown* Genus part of the *Lachnospiraceae* family, C*ryptobacteroides*, *Limimorpha* and *UBA4334* collectively compromising over 41% of relative abundance across all feed treatments. Using UpsetR analysis (Fig. 8) the distribution of genera occurrences across different feed treatment communities (SIL, BG and TN) identified a total of 182 shared genera (Supplementary Table S6). There were 16,16 and 23 genera specific to feed treatments SIL, BG and TN, respectively (Fig. 8) although, the combined relative abundance of these specific taxa unique to each feed treatment was 0.304%, 0.101% and 0.141% for SIL, BG and TN, respectively (Supplementary Table S7).

**Table 5:** Univariate analysis of relative abundance (%) of top ten most abundant genera across all feed treatments

	Treatment				
Genus	SIL	BG	TN	s.e.m	P-value
Other	41.06	41.36	41.56	0.710	0.960
Lenti-01	2.35	1.80	3.06	0.605	0.430
UBA3636	1.77^a^	3.04^b^	2.70^b^	0.181	**
UBA1067	3.44	2.22	2.76	0.246	0.176
SFMI01	4.57	3.52	3.22	0.328	0.405
UBA4334	4.66	3.24	3.52	0.471	0.547
Limimorpha	3.10^a^	4.01^ab^	5.57^b^	0.348	*
Cryptobacteroides	3.66^a^	5.31^b^	5.06^b^	0.234	**
unknown_f__Lachnospiraceae	5.67	7.14	6.24	0.530	0.512
DSXL01	9.37	7.99	8.71	0.754	0.292
Prevotella	20.35	20.37	17.59	0.697	0.152

Analysis of Variance (ANOVA) and Kruskal Wallis test was performed on the relative abundance of top ten genera measuring the impact of feed treatment on genera relative abundance (%). The code ‘*’ and ‘**’ denotes level of significance *P* < 0.05 and *P* < 0.01 respectively in differences amongst feed treatment

SIL, Silage feed treatment; BG, Beagle feed treatment; TN, Terra Nova Feed treatment; s.e.m., standard error of the mean

**Fig. 9:**
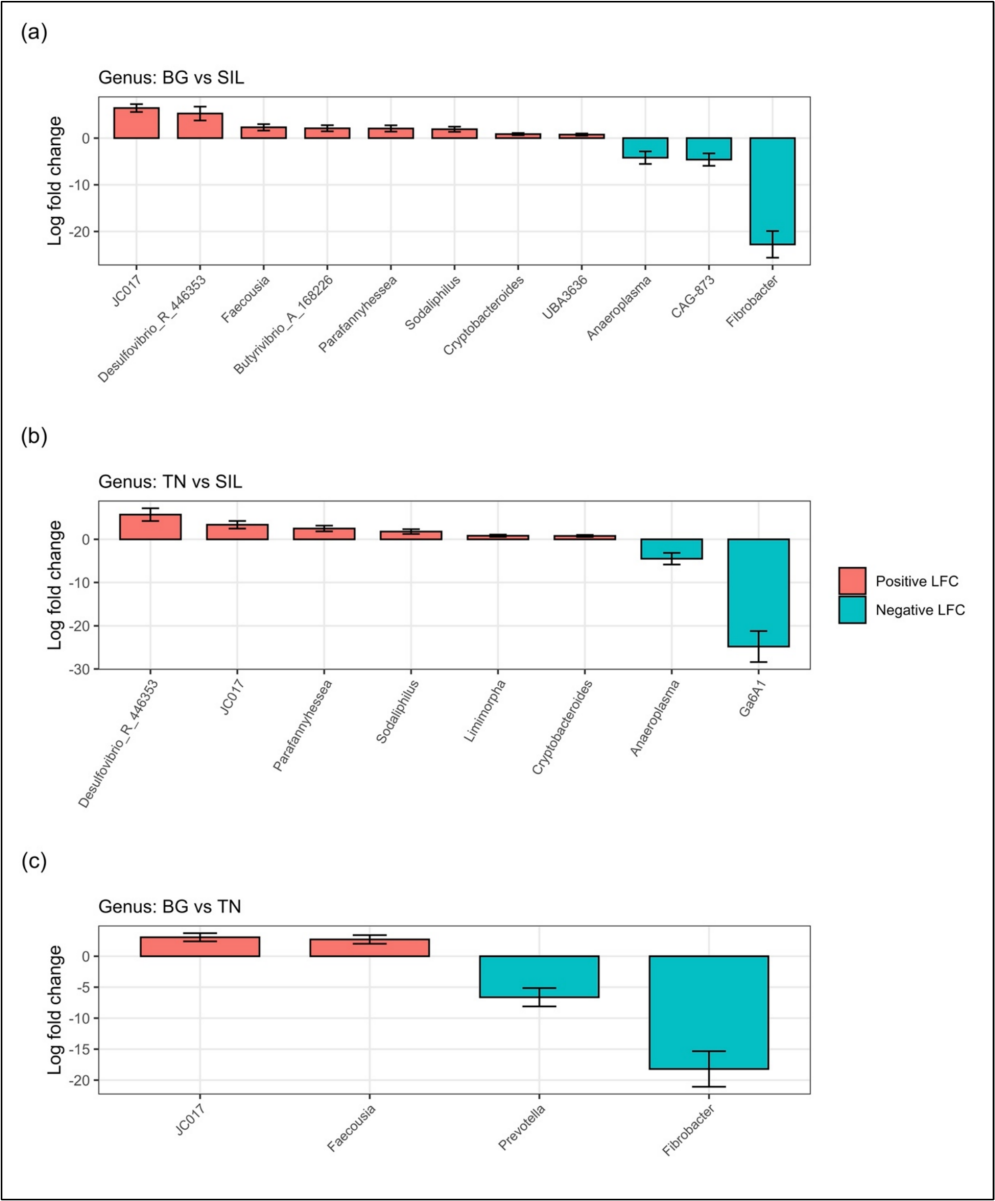
Differential relative abundance across Feed treatment at the genus level. The box plots illustrate the differential relative abundance of ASV at genus level across feed treatment types. Although contrast tests were conducted separately for each sample type: **A** BG vs SIL, **B** TN vs SIL. The x-axis represents the log2 fold changes of significant genera, while the y-axis displays the positive and negative logarithm (base 10) of the adjusted p-values. Red bars represent ASV with a significant positive relative abundance in Feed 1 compared to Feed 2 (P < 0.05), indicating a higher relative abundance in Feed 1. Conversely, blue bars represent ASV with a significantly greater relative abundance in Feed 2 than in Feed 1 (P < 0.05), indicating a greater relative abundance in Feed 2. A Likelihood Ratio Test was used to assess the effect of global parameters, and the Wald test was applied to identify significant genus differences between Feed 1 and Feed 2 for each sample type. Each graph title specifies the overall contrasts and sample types being analysed

Therefore, to investigate the differences within the feed treatment microbial communities, contrast analysis was performed. Figure 9 identifies significant differences (*P* < 0.05) in the relative abundance of various genera between feed treatments with Table 6 showing the Log2Fold change. In the contrast between BG vs SIL, significant genera with a higher relative abundance in BG included: *JC017*, *Desulfovibrio-R-446353, Faecousia, Butyrivibrio-A-168226, Parafannyhessea, Sodaliphilus, Cryptobacteroides* and *UBA3636*. Whereas genera that had a significantly higher relative abundance in SIL included: *Anaeroplasma, CAG-873* and *Fibrobacter*.

**Table 6:** Significant differential relative abundance across feed treatments at the genus level

Genus	Base Mean	log2Fold Change	lfcSE	Stat	P-value	P-adj	Symbol
*BG vs SIL*							
JC017	6.47	6.44	0.84	7.69	0.000	0.000	***
Desulfovibrio_R_446353	6.04	5.26	1.48	3.56	0.000	0.014	*
Faecousia	11.81	2.30	0.69	3.33	0.001	0.016	*
Butyrivibrio_A_168226	10.91	2.10	0.65	3.23	0.001	0.020	*
Parafannyhessea	23.10	2.06	0.67	3.08	0.002	0.026	*
Sodaliphilus	39.10	1.90	0.55	3.44	0.001	0.014	*
Cryptobacteroides	101.66	0.85	0.23	3.68	0.000	0.012	*
UBA3636	81.48	0.75	0.23	3.21	0.001	0.020	*
Anaeroplasma	14.37	-4.19	1.34	-3.13	0.002	0.024	*
CAG-873	4.27	-4.60	1.33	-3.47	0.001	0.014	*
Fibrobacter	18.01	-22.76	2.86	-7.97	0.000	0.000	***
*TN vs SIL*							
Desulfovibrio_R_446353	6.04	5.73	1.48	3.88	0.000	0.013	*
JC017	6.47	3.38	0.88	3.85	0.000	0.013	*
Parafannyhessea	23.10	2.51	0.67	3.77	0.000	0.013	*
Sodaliphilus	39.10	1.80	0.55	3.26	0.001	0.044	*
Limimorpha	129.77	0.84	0.26	3.24	0.001	0.044	*
Cryptobacteroides	101.66	0.79	0.23	3.41	0.001	0.041	*
Anaeroplasma	14.37	-4.49	1.35	-3.34	0.001	0.044	*
Ga6A1	0.72	-24.80	3.60	-6.89	0.000	0.000	***
*BG vs TN*							
JC017	6.47	3.06	0.66	4.63	0.000	0.000	***
Faecousia	11.81	2.71	0.69	3.90	0.000	0.010	*
Prevotella	5.91	-6.62	1.48	-4.47	0.000	0.001	**
Fibrobacter	18.01	-18.20	2.87	-6.34	0.000	0.000	***

The table lists the significant genera and there corresponding log2FoldChange displayed in Fig. 8 for three different feed treatment interactions: BG vs SIL, TN vs SIL and TN vs BG. The code ‘*’, ‘**’ and ‘***’ denotes level of significance *P* < 0.05, *P* < 0.01 and *P* < 0.001 respectively in differences amongst feed treatment

Base mean, the average normalised genus abundance across all samples; log2FoldChange, Log base twofold change of the genus between the two feed treatments; lfcSE, standard error of Log2FoldChange; stat, test statistic for Wald test used to assess significance of differential Abundance; P-value, p-value of Wald test; P-adj, Adjusted p-value which corrects for multiple testing using the Benjamini–Hochberg procedure; Symbol, denotes level of significance

In the contrast between TN and SIL, genera with a significantly higher relative abundance in TN included: *Desulfovibrio-R-446353, JC017, Parafannyhessea, Sodaliphilus* and *Cryptobacteroides*. While *Anaeroplasma* and *Ga6A1* had a significantly higher abundance in SIL.

In comparison between the two CT-containing treatments, BG had a significantly higher abundance of *JC017* and *Faecousia* genera relative to TN. While, TN had a significantly greater abundance of *Prevotella* and *Fibrobacter* relative to BG.

**Fig. 10:**
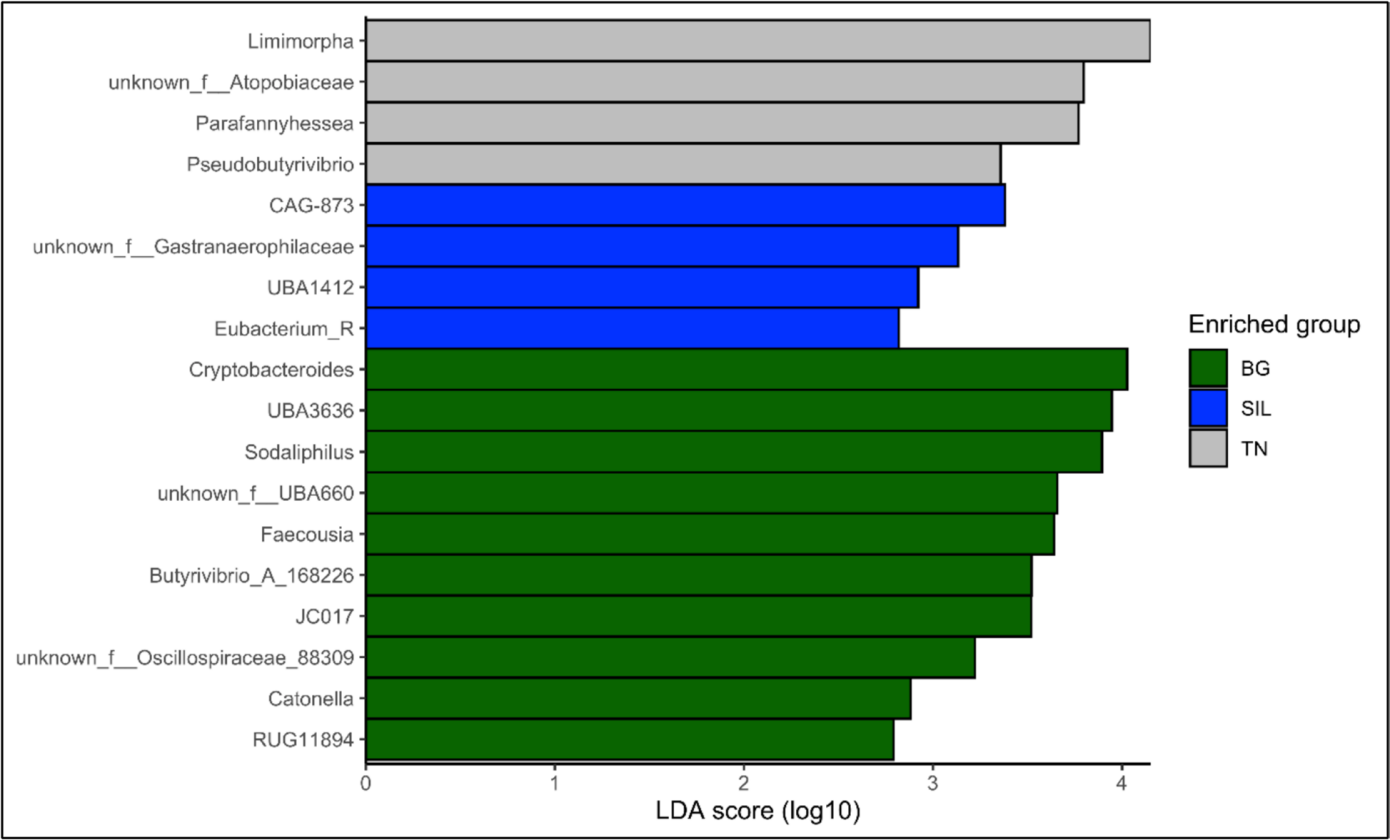
Linear discriminant analysis (LDA) scores of the genera in the rumen of ewe hoggets fed SIL, BG and TN. LDA scores > 2 show genera significantly (P < 0.05) more abundant according to different feed treatments

Linear discriminant analysis (LDA) effect size (LEfSe) was also used to assess differentially abundant genera between feed treatments set a *P* < 0.05. To highlight the effect size of the differences between genera a threshold of two was used. For SIL, significantly abundant genera (*P* < 0.05; Fig. 10) included: *CAG-873*, unknown genus from Family *Gastranaerophiliceae*, *UBA1412* and *Eubacterium_R*. The differential abundance of ten genera were significant for BG (*P* < 0.05) and included: *Crypobacteroides, UBA3636, Sodaliphilus,* unknown genera of family *UBA660*, *Faecousia, Butyrivibrio-A-168226, JC017,* unknown genera of family *Oscillospiraceae-88309*, *Catonella* and RUG11894. While for TN, *Limimorpha,* unknown genera of Family *Atopobiaceae, Parafannyhessea, Pseudobutyrivibrio* and *CAG-873* genera had significant differential abundances.

### 2.5 Effect of diet on selected rumen metabolite concentration

**Fig. 11:**
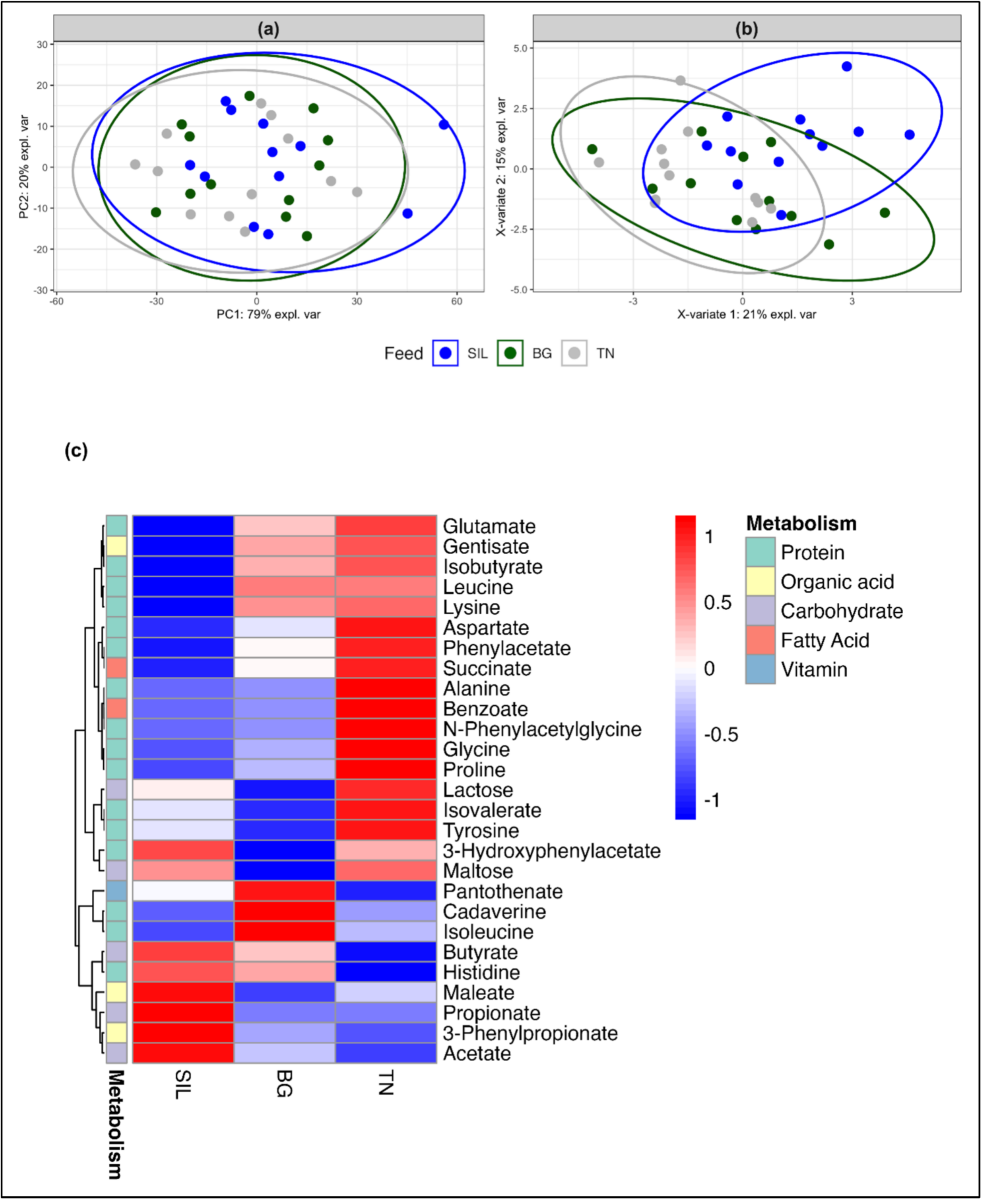
Exploratory analysis of Rumen metabolites from ewe hoggets fed three different feed treatments of differing condensed tannin content. **A** PCA plot of rumen metabolites grouped according to feed treatment. PC1 and PC2 account for 79% and 20% respectively of the total variation. **B** PLSDA plot of rumen metabolites grouped according to feed treatment. X-variate 1 and X-variate 2 account for 21% and 15% respectively of the variation. **C** Heatmap of different rumen metabolites concentrations classified according to the main type of metabolism they are involved for the three different feed treatments of differing condensed tannin content. Hierarchical clustering analysis of the average metabolite concentration was calculated using Euclidean distance resulting in a scale of + 1 (Red: higher concentration) to -1 (Blue: lower concentration)

Following initial exploratory metabolite analysis, no significant differences were observed between NMR buckets of metabolites from different feed treatments. Nevertheless, twenty-seven metabolite concentrations (Supplementary Table S8) were selected and extracted from the Chemonx spectral software analysis based on specific protein and fibre metabolic pathways that have potential to be affected by CT inclusion in the diet. This included compounds involved in protein, carbohydrate, fatty acid, organic acid and vitamin metabolism. The pathways and the main metabolic classification that the selected metabolites are individually involved in were assigned using the Kyoto Encyclopaedia of Genes and Genomes (KEGG) and displayed in Supplementary Table S9. Visualisation of rumen metabolites analysis by feed treatment are shown in Fig. 11. The PCA scores plot (Fig. 11a) showed no clear separation by feed treatment with PC1 and PC2 accounting for 79% and 20% of the total variation in selected rumen metabolites. The PLS-DA, a supervised model for predictive and descriptive modelling in addition to discriminative variable selection, was used. In the PLS-DA scores plot (Fig. 11b) groups were non discriminated and showed no separation according to feed treatment.

Univariate analysis showed no significant differences between ruminant metabolites of different feed treatments (Supplementary Table S8). To visualise any relationships and differences in the average concentrations of rumen metabolites among feed treatment, hierarchical cluster analysis (HCA) using average linkage and Euclidean distance was used and visualised using a heat map (Fig. 11c). Based on visual trends observed in the heatmap analysis metabolites associated with in protein metabolism appeared most abundant in the TN feed treatment and decreased in order by BG and SIL. For carbohydrate metabolism, SIL showed a visually higher concentration of related metabolites followed by descending order of TN and BG. Organic acid metabolism also appeared elevated in SIL feed treatment with no clear visual differences between CT treatments. Concentration of fatty acid metabolites seemed highest in TN and descended in the order with BG followed by SIL. Lastly, vitamin metabolism showed a visual trend of higher concentration of metabolites in BG and decreased accordingly in the order of TN followed by SIL. These patterns, while not statistically significant, suggest potential treatment-related shifts in metabolic profiles.

### 2.6 Correlations of significant rumen microbiome taxa and fermentation parameters

**Fig. 12:**
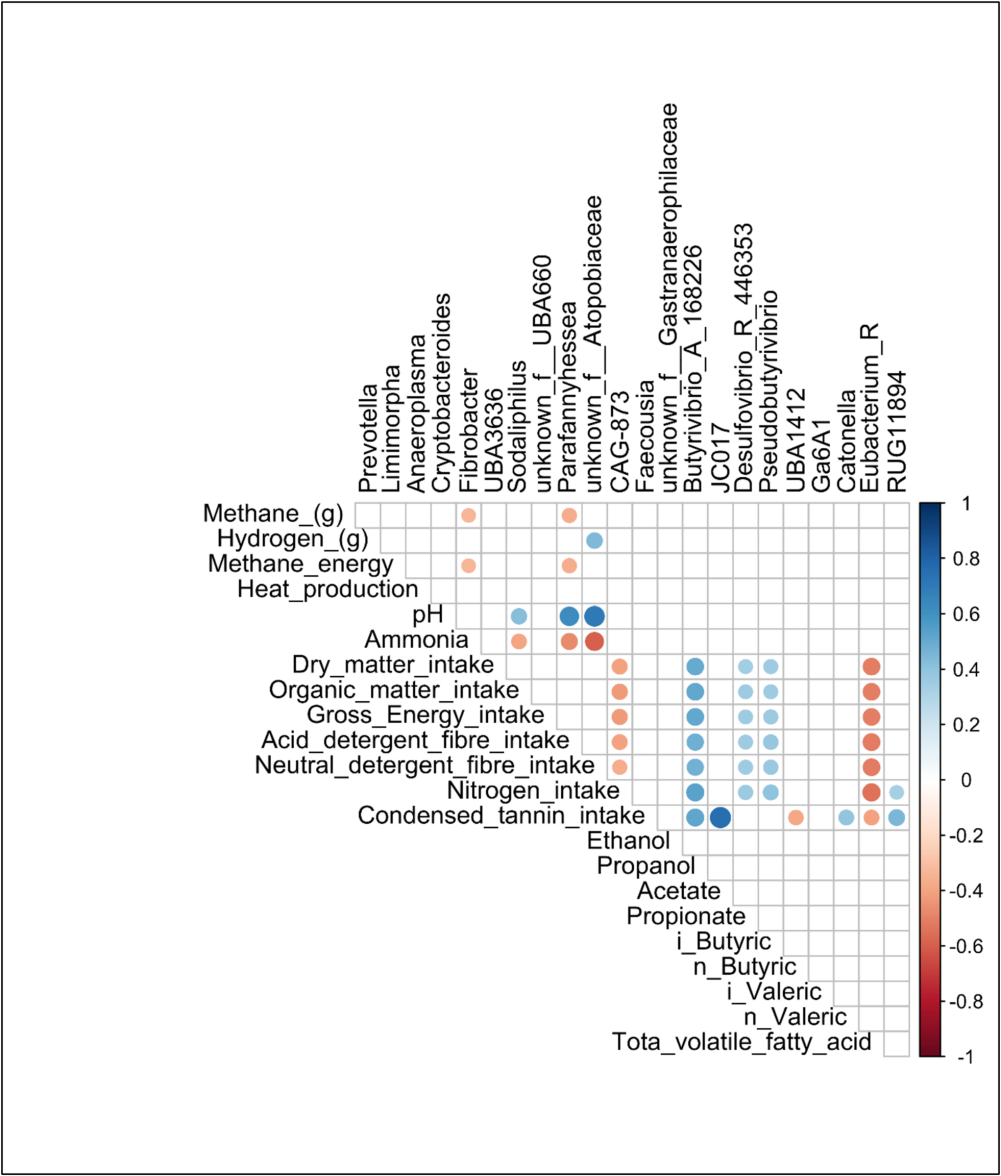
Correlation plot between significant taxa and rumen fermentation parameters. Correlation was conducted using Spearman’s correlation and only significant correlations (P < 0.05) are displayed. Correlations are coloured according to the correlation result with + 1 correlations coloured dark blue and -1 correlations coloured dark red

Spearman’s rank correlation between significantly different rumen taxa at genus level and fermentation parameters are shown in Fig. 12. Only significant correlations (*p* < 0.05) are displayed with the corresponding correlation value assigned a colour from a range of + 1 (dark blue) to − 1 (dark red). Genera *Fibrobacter* and *Parafannyhessea* were both negatively correlated to ruminal methane production and methane energy whereas unknown genera of family *Atopobiaceae* was positively correlated to ruminal hydrogen production. Regarding ruminal NH_3_ production genera *Sodaliphilus, Parafannyhessea* and unknown genera of family *Atopobiaceae* were negatively correlated.

Genera *CAG-873* and *Eubacterium-R* were both negatively correlated to nutrient intake parameters (DMI, OMI, GEI, ADFI and NDFI) while *Butyrivibio_A_168226*, *Desulfovibrio_R_446353* and *Pseudobutyrivibrio* were positively correlated to these nutrient intake parameters in addition to nitrogen intake. While genera *RUG11894* was also positively correlated to nitrogen intake, *Eubacterium_R* was strongly negatively correlated to nitrogen intake. Finally, condensed tannin intake was positively correlated to *JC017*, *Butyrivibio_A_168226*, *Catonella* and *RUG11894*, while *UBA1412* and *Eubacterium-R* were both negatively correlated condensed tannin intake.

## Discussion

### 3.1 Freezing Preservation reduced the condensed tannin content of Willow

Freezing as a preservation method for willow leaves had negative impacts on CT content. Distribution of CTs can be free-unbound, protein-bound or fibre-bound mainly located in the vacuole of the plant cell with some found in the leaf epidermis [17, 18]. However, at temperatures below their freezing point, plant cell vacuoles can lyse in the cytoplasm forming smaller vacuoles [19]. It is the lysis of these vacuoles that causes the release of CT into the plant cytoplasm, exposing these CTs to cytoplasm-based oxidation–reduction processes, permitting their modification through oxidation resulting in the decreases of CT content seen in the present study. Similarly, another study assessed the impact of ensiling and drying to make hay of sainfoin and sulla. It was observed that polyphenol, total CT content, and CT fractions were reduced more in the silage than in the hay, compared to the fresh forages of sainfoin and sulla. Lastly, this reduction in CT content was also observed when the analysis of oxidised fractions of CTs by thiolysis showed a 20–40% reduction in native procyanidin structures, indicating that procyanidin structures had been extensively altered by oxidation, leading to the formation of new oxidative linkages resistant to thiolytic degradation [20].

In the present study, upon visual appearance, TN possessed a much darker colour post freezing than BG implying TN suffered more oxidation than BG [21]. This observation is supported by the fact purified TN CTs from frozen samples exhibited a much greater proportion of A-type interflavan linkages present compared to purified BG CTs from frozen samples (Table 2) and the fact that purified BG and TN CTs from fresh samples were void of A-type interflavan linkages. These A-type linkages result from oxidation–reduction reactions taking place in the B-ring of any of a string of catechin and epicatechin units in CTs giving rise to a highly reactive species called quinomethides [22]. When the quinomethides react intramolecularly, this can lead to A-type linkages between adjacent flavanol units. Alternatively, if the quinomethide reacts with a neighbouring tannin molecule [23] or other biomolecule, the flavan-3-ol subunit which formed the crosslink no longer has the capability to provide the anthocyanidin colour typically displayed in the HCl-butanol reaction. Therefore, the rise in the proportion of A-type interflavan linkages in the structure of CTs corresponds to a loss in CT content and is exactly the case when BG and TN CTs are frozen. However, the factors contributing to the frozen TN CTs having a greater proportion of A-type and lower CT content relative to frozen BG CTs are unknown and highlights freezing had different effects on willow varieties. Nevertheless, it resulted in BG having a higher CT content to bind to protein/fibre and other plant molecules to impact on rumen fermentation suggesting it should have more of an effect relative to TN [24]. As a final note, A-type linkages promote bending of the linear CTs containing all B-type interflavan linkages which would limit the functional surface area from which a CT can readily interacting with protein surfaces [20]. Thus, oxidation of CTs leads a reduction of CT content in plant materials in addition to reducing their effectiveness to bind proteins.

### 3.2 Condensed tannins impacted rumen microbiome

The combined fibre intake (ADFI and NDFI) was significantly higher for BG and TN compared to SIL without reducing FDMI and overall TDMI. This contrasts with the literature where fibre has been negatively correlated to TDMI [25]. Nevertheless, fibre digestion of BG and TN appears to have been reduced as indicated by the significant decreases in acetate production relative to SIL. At high concentrations, excess CT, can depress fibre digestion through complexing with lignocellulose preventing microbial digestion or directly inhibiting cellulolytic bacteria and activities of fibrolytic enzymes or both [26–28]. Cellulolytic activity is driven by a varied bacterial community, with the main members belonging to the *Ruminococcus* and *Fibrobacter* genera [29–31].

In the contrast between BG and SIL, the prevalence of *Fibrobacter* was significantly much greater in SIL. Therefore, at a CTI of 1.1% DM, inhibition of *Fibrobacter* has likely occurred. This matches up with other studies which disclose CT have a direct inhibitory effect on hemicellulolytic, endoglucanase and proteolytic enzymes of *Fibrobacter, Butyrivibrio, Ruminobacter* and *Streptococcus* bacteria [7]. Furthermore it was found that a blend of tannins increased *Ruminococcaceae* and other members of phylum *Firmicutes* while inhibition of genera *Prevotella* and *Fibrobacter* occurred [32]. Regarding the contrast between TN and SIL, no clear cellulolytic bacteria differences in abundances occurred despite TN having a reduced ruminal acetate production and its CTI was 0.1% TDMI. Nevertheless, the effect of CT treatments (BG and TN) on inhibiting fibre digestion can be observed from the rumen metabolomic results. From the heatmap generated on this data (Fig. 11c) it can be observed that metabolite concentrations involved in carbohydrate metabolism showed a trend of being higher in SIL and reduced in the descending order of TN and BG. This trend could suggest less fibre was broken down into volatile fatty acids in the rumen by BG and TN which could have been used for carbohydrate metabolism despite these treatments having a higher fibre intake relative to SIL.

CT are well known to bind to proteins in the rumen decreasing their degradation to NH_3_ production and increasing the protein outflow to the abomasum were a pH drop causes the CT and protein to dissociate and be available for uptake or excretion [33]. If the dissociation successfully takes place in the abomasum, inclusion of CT can have benefits for livestock production. In this study no differences were observed in ruminal NH_3_ production between CT-containing treatments and SIL. In addition to binding to protein, CT can decrease the activity of bacterial proteinases [34] and can decrease the growth of proteolytic bacteria. Proteolytic bacteria play a key role in protein metabolism in the rumen primarily breaking proteins into amino acids using endoproteases (both cell bound and secreted) and peptidases [35]. *Streptococcus bovis* has been identified as highly proteolytic bacterium while *Prevotella ruminocola* are involved in peptide breakdown which is faster than amino acid degradation [36]. Despite BG and TN having a higher NI, no differences were observed in ruminal ammonia concentrations relative to SIL. Consequently, no differences in the relative abundance of proteolytic bacteria were observed in the contrasts between BG and TN with SIL. In other studies, CTs from *Lotus corniculatis* were shown to modify individual proteolytic bacterial populations in the rumen of sheep and resulted in a decline of *Clostridium proteoclasticum, Butyrivibrio fibrisolvens*, *Eubacterium sp. C12b*, and *Streptococcus bovis* [37]. However, it is important to note that some ruminal bacteria can ferment proteins and peptides in the presence of CT [4], while other studies have shown proteolytic bacteria such as *Prevotella bryantii* showed higher resistance to CT [34, 38]. Although non-significant concentration of metabolites involved in protein metabolism showed a trend of being highest in CT-containing treatments TN and BG and lowest in SIL, which could indicate that less protein was degraded and converted to ruminal NH_3_ due to the presence of CT and their assumed ability to bind with protein in the rumen. From the correlation results, the genera *Sodaliphilus*, *Parafannyhessea* and unknown genus of family *Atopobiaceae* were negatively correlated to ruminal ammonia concentration. All these genera were found in the CT-containing BG and TN treatments suggesting a possible link.

Regarding the contrast between BG and TN, *Prevotella* and *Fibrobacter* had a significantly greater abundance in TN relative to BG. This is unsurprising due to the BG having a greater CT content relative to TN (10 × greater). Additionally, the structure of BG CTs had a significantly greater PD content (BG = 62.7%; TN = 5.42%). A greater proportion of PD over PC results in a greater number of hydroxyl groups in the CT structure, which increases the potential for hydrogen bond interactions resulting in greater protein binding and precipitation capacity [16]. Several studies have shown that the complexing capacity of CTs rises with an increasing PD content [39–42].

Positive correlations of significant genera in BG and TN (*JC017, Butyrivibrio_A_168226, Catonella* and *RUG11894* with CTI suggest the taxa could play a role in nutrient degradation when CTs are added to the diet of ruminants. While most of their functions are relatively unknown, *Butyrivibrio* bacteria have been shown to have widespread machinery for polysaccharide degradation such as carbohydrate-active enzymes (CAZymes) (Palevich et al., 2019; Pidcock et al., 2021)*.* With CT addition to the diet shown to decrease fibrolytic bacteria, this observation demonstrates the metabolic plasticity and resilience of the rumen microbiome to dietary changes.

## Conclusion

While CT from BG and TN had no significant impact on reducing ruminal CH_4_ and NH_3_ production, these treatments resulted in a greater forage and total dry matter intake responsible for increased nitrogen and fibre (NDF and ADF) intake relative to silage. At a CTI of 1.1% and 0.1% for BG and TN, respectively, significant differences occurred in rumen microbiome taxonomy at the genus level and composition with BG reducing *Fibrobacter* abundance. While no significant differences were observed in the rumen metabolites between treatments, metabolites involved in carbohydrate metabolism tended to be lowest in CT treatments, suggesting inhibition of fibre degradation, which aligns with the significantly lower ruminal acetate production in BG and TN. Moreover, metabolites involved in protein metabolism tended to be higher in TN and BG compared to SIL, suggesting that CT were binding to protein in the rumen, preventing degradation to NH_3_. The findings indicate that feeding willow CTs enhanced feed intake, altered rumen microbiome composition, and protein metabolism, but did not affect growth. While a reduction in CH_4_ was not observed, this study highlights the potential of willow to modify ruminant nutrition.

## Material and methods

### 5.1 Animals and housing

The animal study took place at the Agri-Food and Biosciences Institute, Northern Ireland (54.4518° N, 6.0748° W, and 112 m altitude). Twelve ewe hoggets (6 Texel x Mule and 6 Suffolk x Mule) were used in a three (treatment) x three (period) Latin square design experiment with a single period lasting 21 days and the overall experiment lasting nine weeks. Hoggets aged approximately 13 months and mean mass of 56.292 kg (s.d. 5.176 kg) were allocated to three treatment diets balanced according to sire breed, dam breed and body weight. Each treatment group was composed of two Texel x mule and two Suffolk x mule hoggets. Throughout the experiment, during each period, hoggets were housed in a single group on plastic slats with access to water and treatment diets through the Controlling and Recording Feed Intake (CRFI) system (CRFI, BioControl, Poland, Europe). During the experiment, the initial two weeks were counted as the adaptation phase and the third week was the measurement phase.

### 5.2 Willow fodder harvesting and preservation

Two varieties of willow, *Beagle* (BG) and *Terra Nova* (TN), were selected for experimentation based on palatability and CT content and structure [16]. Willow fodder consisted of leaves only and harvesting commenced in early May 2022 until mid-July 2022 at the same location of the animal study. Fodder was stripped from willow branches by hand and shredded (up to 4 cm) using a BOSCH AXT 25 D 2500W Shredder (BOSCH, Milton Keynes, United Kingdom). Weighed amounts of willow fodder, according to daily DM requirements, were compressed into plastic bags and bags were evacuated using a Delfin 202 DS Industrial vacuum (Delfin, Italy) to generate anaerobic conditions before the bag was sealed air-tight. Each fodder bag was spot frozen at -80 °C for five days and then moved to − 20 °C for the remainder of storage. Each bag was removed 24 h before feeding and allowed to thaw at 3–5 °C.

### 5.3 Treatments

During a two-week pre-experimental period, hoggets were offered grass silage (SIL) plus the concentrate (0.18 kg) administered through a GreenFeed system (GF system, C-Lock, Inc., South Dakota, USA). During the experiment, all hoggets were offered a set dry matter intake (DMI) to correspond to maintenance energy requirements calculated according to AFRC [43]. Treatments examined (BG, SIL, TN) differed in willow fodder inclusion and tested two different willow varieties at a set 20% inclusion rate on a DM basis. The BG and TN diet consisted of 20% willow fodder and 80% grass silage on a DM basis and were weighed out accordingly and mixed using a Belle Premier 200XT mixer (Belle, Derbyshire, UK). The SIL diet consisted of 100% grass silage and was used as the control. All treatments were supplemented with 0.18 kg of concentrate (Intensive lamb pellet; Fane Valley Feeds, Northern Ireland, Supplementary Table S1).

### 5.4 Feed sampling and analysis

Daily samples of BG, SIL and TN were collected and combined to obtain a representative sample of the diet. The collected materials were oven-dried at 60 °C (VWR International, Radnor, Pennsylvania, USA) until a constant mass was achieved and milled to a particle size of ≤ 1 mm (Christy & Norris Lab Mill, Christy Turner Ltd, Suffolk, UK) and stored at room temperature. The samples were analysed for dry matter (DM), ether extract (EE) and ash according to official AOAC methods [44]. Acid detergent fibre (ADF) and neutral detergent fibre (NDF) were determined according to Goering and Van Soest [45] and were performed on the ANKOM 220 Fibre Analyser (ANKOM Technology Corporation, New York, USA) with sodium sulphite and heat stable α-amylase. Nitrogen content in the samples was analysed via the Dumas method [46], with the Leco Protein Vario Max CN analyser (Elementar Analysensysteme, GmbH, Hanau, Germany) and crude protein (CP) was calculated using N × 6.25. Gross energy (GE) content was determined by AFBI Hillsborough Analytical Services using an isothermal automated bomb calorimeter (PARR, Instrument Model 6300, UK). An additional weekly fresh sample of SIL was analysed using near infrared spectroscopy (NIRS) for DM, CP, ADF, NDF, water soluble carbohydrate (WSC; %DM) and metabolisable energy (ME; MJ/kg DM).

Concentrate (C) samples were collected three times during each period from both GF systems. Samples were analysed for DM, GE, N, CP, Ash, EE, NDF and ADF determinations as outlined above and starch concentrations were determined using a total starch assay kit (Megazyme International Ireland Ltd, Wicklow, Ireland; McCleary et al. 1994).

### 5.5 Condensed tannins

Weekly forage samples were collected for the determination of their CTs content from three different collection points, pooled and oven dried at 30 °C for 96 h, passed through a 1 mm sieve and stored in the dark. The *Salix* Terra Nova and Beagle CT reference standards were obtained following the procedures described in Brown et al. and Naumann et al. [47, 48].

Ground *Salix* Beagle leaf material (50 g) was placed in a 1000 mL Erlenmeyer flask equipped with a magnetic stir bar and diluted with acetone/water (7:3, 500 mL). The mixture was rapidly stirred for 30 min and then filtered through a glass-sintered funnel equipped with a filter paper (Reeve Angel, grade 202). The residue was returned to the Erlenmeyer flask and stirred with fresh acetone/water (7:3, 500 mL) and filtered two additional times. The combined three acetone/water extracts were concentrated under reduced pressure (rotary evaporation) at ≤ 40 °C to remove the acetone and the resulting aqueous layer was stirred with ethyl acetate (400 mL) overnight. The aqueous layer was separated using a separatory funnel and stirred a second time with ethyl acetate (200 mL) for about 2 h. The aqueous layer was separated and placed under reduced pressure (rotary evaporation) at ≤ 40 °C to remove any traces of ethyl acetate and then freeze-dried to give 11.190 g of extract. This extract was diluted with methanol/water (1:1, 100 mL) and Sephadex LH-20 (GE Healthcare, Uppsala, Sweden) was added in small portions while stirring with a spatula, until the mixture reached the consistency of wet sand (29.679 g of Sephadex LH-20 added). The CT-laced resin was transferred to a 500 mL sintered-glass funnel equipped with a filter paper (Reeve Angel, grade 202). The resin was suspended in methanol/water (1:1, 200 mL), allowed to stand for ~ 5 min and then vacuum filtered. This methanol/water washing of the resin was repeated 14 additional times. The resin was then suspended in acetone/water (7:3, 200 mL), allowed to stand for ~ 5 min and then vacuum filtered. This acetone/water washing of the resin was repeated three additional times. The four acetone/water washings were combined and concentrated under reduced pressure (rotary evaporation) at ≤ 40 °C to remove the acetone and freeze dried to give 1.87 g of an off-white solid, sufficiently pure to serve as the *Salix* Beagle CT reference standard in this study.

The *Salix* Terra Nova CT reference standard was obtained a similar manner with the following changes: 50 g of *Salix* Terra Nova leaf material**,** extraction solvent (3 × 7:3 acetone/water, 500 mL); amount of ethyl acetate used to extract non-polar organic, 1st extraction 450 mL, 2nd extraction 225 mL; material amount of initial dried extract obtained, 11.81 g; volume of 1:1 methanol/water used to dissolve extract (180 mL); Sephadex LH-20 mass used, 54.5 g; 1:1 methanol/water washes of Sephadex LH-20 (15 × 250 mL); 7:3 acetone/water washes of Sephadex LH-20 (4 × 250 mL); mass of freeze-dried, purified CT from *Salix* Terra Nova was 0.613 g. The ^1^H − ^13^C HSQC NMR spectrum of this sample indicated that a purer CT sample was required. Thus, 578 mg of the residue was dissolved in methanol/water (1:1, 30 mL) and Sephadex LH-20 (GE Healthcare, Uppsala, Sweden) was added in small portions while stirring with a spatula, until the mixture reached the consistency of wet sand (7.477 g of Sephadex LH-20 added). The CT-laced resin was transferred to a 125 mL sintered-glass funnel equipped with a filter paper (Reeve Angel, grade 202). The resin was suspended in methanol/water (1:1, 50 mL), allowed to stand for ~ 5 min and then vacuum filtered. This methanol/water washing of the resin was repeated 14 additional times. The resin was then suspended in acetone/water (7:3, 50 mL), allowed to stand for ~ 5 min and then vacuum filtered. This acetone/water washing of the resin was repeated three additional times. The four acetone/water washings were combined and concentrated under reduced pressure (rotary evaporation) at ≤ 40 °C to remove the acetone and freeze dried to give 351 mg of an off-white solid, sufficiently pure to serve as the *Salix* Terra Nova CT reference standard in this study.

^1^H, ^13^C, and ^1^H − ^13^C HSQC NMR spectra for the purified *Salix* Terra Nova and Beagle CT were recorded at 27 °C on a BrukerBiospin DMX-500 (^1^H 500.13 MHz, ^13^C 125.76 MHz) instrument equipped with TopSpin 3.5 software and a cryogenically cooled 5 mm TXI ^1^H/^13^C/^15^N gradient probe in inverse geometry. Spectra were recorded in DMSO-*d*_*6*_ and were referenced to the residual signals of DMSO-*d*_*6*_ (2.49 ppm for ^1^H and 39.5 ppm for ^13^C spectra). For ^1^H − ^13^C HSQC experiments, spectra were obtained using the standard Bruker pulse program “hsqcetgpsi”.

For sequential CT content determinations, the HCl-butanol-acetone-iron (HBAI) assay was used [49]**.** The reaction medium was prepared fresh daily on a per 200 mL basis by first dissolving 333 mg of ammonium iron (III) sulphate dodecahydrate in 11 mL of concentrated HCl (37%, w/v) and then adding 93 mL of n-butanol, followed by 96 mL of acetone with stirring; then 13.5 mL of this solution was mixed with 1.5 mL of acetone − water in all tubes, this yielded a final reaction medium formulation similar to that used in the direct assay [50]. CT content determinations were performed in triplicate runs on different days and determinations are averages of triplicate analyses. The sample, previously ground to pass through a 1 mm screen, was briefly ball-milled for 2 min to provide a more homogenous sample. Approximately 20 mg (to the nearest tenth of a mg) of *Salix* Terra Nova and Beagle samples were weighed into Teflon-capped 30 mL capacity thick-walled, screw-capped glass centrifuge tubes (e.g., Kimble 45,600–30). A mixture of acetone–water (7:3, 15 mL) was added to each of the tubes, the tubes were capped and subjected to sonication (Bronsen 8510 sonicator) for 45 min with gentle mixing every 15 min to resuspend the tissue. The tubes were then centrifuged at 6000 × g for 20 min at room temperature, and extract supernatants were completely decanted without disturbing insoluble residue pellets. Subsamples of the decanted acetone − water supernatant (1.5 mL) were transferred to Teflon-capped 25 mL capacity thick-walled glass tubes. Next, 1.5 mL of 7:3 acetone–water was added to insoluble residues in centrifuge tubes and to CT standards (0.0, 0.125, 0.250, 0.375, 0.500 and 0.625 mg) in Teflon capped 25 mL capacity thick-walled glass tubes. The HBAI assay solution (13.5 mL) was added to all tubes. The screw caps were added to the test tubes and the tubes were heated in an aluminium block at 70 °C for 3 h. Every 15 min the tubes were removed and briefly vortexed to ensure proper mixing during the reaction. After cooling to room temperature over approximately 1 h, 2 mL aliquots were removed from each of the triplicate runs, placed in 2 mL conical polypropylene-copolymer microcentrifuge tubes, sealed with screw caps, and centrifuged for 5 min at 10 000 g. After centrifugation, the clarified supernatants were scanned with a Shimadzu UV-2600 spectrophotometer (Shimadzu Scientific Instruments, Columbia, USA) using the Shimadzu UVProbe version 2.43 software package from 400 to 600 nm, and the maximal absorption at λ_max_ of the anthocyanidin peak was recorded. The absorbance data was corrected for the small dilution factor of the reference standards and for the purity of the reference standard.

### 5.6 Animal measurements

Quantities of feed intake per hogget were measured using the CRFI system allowing total dry matter intake (TDMI) calculation. Individual body weights were calculated weekly at the same time during the trial with daily liveweight gain (DLWG) calculated as the regression of BW over time. GreenFeed (GF, C-Lock, Inc., South Dakota, USA), a portable open circuit head system was used to measure O_2_ consumption and CO_2_, CH_4_ and H_2_ production during the full animal experiment. A pelletised C bait (Intensive lamb pellet, Fane Valley Feeds, UK) was offered to entice the hoggets to visit the GF system. The system was configured to allow hoggets to visit at minimum 5-h intervals. During each visit, ewe hoggets were given 8 drops of 0.006 kg bait C every 40 s. The average C bait intake from the GF system was 0.18 kg DM/d. The system also recorded muzzle position during the visits and data with inappropriate muzzle positions were removed.

### 5.7 Rumen fluid sampling and analysis

Before feeding on the last day of the period (day 28), rumen fluid (RF) samples (30 mL) were collected using an oral stomach tube, of length 1.2 m and width of 0.01 m connected to an adjustable vacuum pump. All RF was filtered through cheesecloth removing saliva and feed particles followed by immediate recording of pH values. After this, samples were spot frozen in liquid nitrogen and then stored at − 80 °C for ammonia N (NH_3_-N), microbiome and metabolomic analyses.

### 5.8 DNA extraction from rumen fluid samples and 16S RNA gene sequencing

DNA was extracted from RF samples using the QIAGEN DNeasy PowerSoil Pro Kit (QIAGEN, Manchester, United Kingdom) following manufacturing instructions with two additional steps. At the start, 500 µL of RF was centrifuged and during the extraction, the FastPrep-24 instrument (MP Biomedicals, California, USA) was used to aid cell lysis performing three 60-s cycles at a speed of 5.5 m/s, with the tube being placed on ice in the intervals between bead beatings. Thirty-six samples were processed consisting of four samples for each treatment (× 3) across three time points. Four negative controls were conducted and included in the utilisation of only the kit reagents (representing the ‘kitome’). As a positive control, the ZymoBIOMICS Microbial Community Standard (D6300, Zymo Research Corporation) was selected. All extracted DNA underwent nucleic acid quantification determined using a DeNovix DS11-FX Spectrophotometer (DeNovix, Delaware, USA) calculating the concentration and turbidity of the genomic DNA. Circular consensus sequencing (CCS) reads were sequenced by the Queen’s University Belfast Genomics Core Technology Unit (GCTU) using the PacBio Sequel-II (Pacific Biosciences) sequencer. PacBio’s Single Molecule, Real Time (SMRT) sequencing pipeline generated HiFi reads following the manufacturer’s protocols for library preparation and sequencing (PacBio, 2022a b). The 16S RNA HiFi reads were deposited in ENA (https://www.ebi.ac.uk/ena/, uploaded on 26th January 2024) under the accession code ERS17888369/ERA28151579/.

The HiFi reads were analysed using QIIME2 (Quantitative Insights Into Microbial Ecology 2) platform (QIIME 2, version 2023.7.0) [51]. Sequences were denoised using the DADA2 denoise-ccs plugin [52]. The denoising parameters included the primers sequences 27F and 1492R (–p-front AGRGTTYGATYMTGGCTCAG; –p-adapter RGYTACCTTGTTACGACTT), min-length 1,000 and max-length 1,600. Amplicon sequence variants (ASV) generated were then taxonomically classified using the ‘classify-sklearn’ classifier with the full-length pre-trained Naive Bayes classifier based on the Greengenes 2022 database (2022.10.backbone.full-length.nb.qza). Tabled read counts were visualised in the QIIME 2 viewer and exported at the species level (Level 7). Taxonomic assignments that contained the terms “unclassified”, “unidentified”, “group”, or “uncultured” were relabelled as “unknown” to facilitate estimation of the taxonomic assignment yield at each taxonomic level (e.g. d__*Bacteria*, p__*Firmicutes_A*, o__*Clostridia_258483*, c__*Lachnospirales*, f__*Lachnospiraceae*, g__unknown, s__unknown). The “unknown” taxa was then concatenated to its last know classification prior to the calculation of downstream metrics (e.g. d__*Bacteria*, p__*Firmicutes_A*, o__*Clostridia_258483*, c__*Lachnospirales*, f__*Lachnospiraceae*, g__unknown_f__*Lachnospiraceae*).

Data analysis was conducted using R (version 4.4.2) and RStudio (version 4.4.0) with taxonomic data stored in a phyloseq object that was provided as an optional output from the QIIME2 package. Initial exploratory analysis was conducted using the phyloseq package (version 1.48.0). Using the decostand function from the vegan package (version 2.6.6.1), the data was normalised with total sum scaling (TSS). Univariate analysis of relative abundance at phyla level between treatments was performed using the stats package (version 4.4.0). Principal component analysis (PCA) [53] was conducted according to feed treatment using ggplot2 (version 3.5.1). Alpha diversity indices [54] such as richness (Chao1), evenness (Pielou's), and diversity (Inverse Simpson) were computed using the vegan package (version 2.6–8) [55] and displayed in box plots. Beta diversity [56] was visualised using a principal coordinate analysis (PCoA) and computed using the vegan package (version 2.6–8) [55]. Bray–Curtis dissimilarity index between feed treatments was analysed using PERMANOVA [57] and computed using the adonis2 function in the vegan package. Stacked bar plot of taxonomy and composition were visualised using ggplot2 [58]. The occurrence distribution of genera across all samples were determined using the UpsetR package [59] (version 1.4.0). Relative abundance of taxa according to feed treatment and visualisation using a heat map were conducted using the phylosmith package (version 1.0.7) [60]. Differential relative abundance across feed treatments were conducted using DESeq2 (1.44.0) through which raw count data were normalised and evaluated using the likelihood ratio test (LRT) for contrasts between feed treatments [61]. Furthermore, linear discriminant analysis (LDA) [62] effect size (LEfSe) was used to identify the most differentially abundant taxa among treatments in R using the microbiomeMarker package [63] (version 1.10.0).

### 5.9 Metabolomics based on NMR and data processing

Metabolites were extracted from rumen fluid using a biphasic methanol/chloroform/water method used previously by Southam et al. (2020) [64]. Briefly, 350 µL of rumen fluid was mixed with 788 µL methanol (LC–MS grade, VWR) and vortexed (full power, room temperature, 15 s). Then 150 µL of chloroform (HPLC plus grade, Sigma) and 313 µL of water (LC–MS grade, VWR) were added and the sample was vortexed (full power, room temperature, 30 s) and centrifuged (2,500 g, 18 °C, 10 min). Then, 1047 µL of the resulting supernatant was combined with 500 µL of chloroform and 313 µL of water and vortexed (full power, room temperature, 30 s). All reagents in these steps were under ice cold conditions. Samples were then centrifuged (2,500 g, 18 °C, 10 min), and then incubated at room temperature for five min to allow completion of the biphasic separation. Then 1 mL of the upper phase (containing the polar metabolites) was transferred into a 2 mL microfuge tube and dried in a SpeedVac concentrator (Savant SPD111V230, Thermo Fisher Scientific). Dried samples were stored at − 80 °C until NMR analysis. Prior to NMR analysis, rumen samples were resuspended in 700 uL 10:90 Urine Preparation NMR Buffer (Avance IVDr, Bruker):LC–MS grade water with formic acid (0.5 mM). Samples were incubated at room temperature for 15 min and then centrifuged (20,000-g, 20 °C, 15 min) and 600 uL was loaded into a 5 mm NMR Tube (Bruker).

NMR spectra were acquired on a Bruker IVDr 600 MHz spectrometer (Bruker, Billerica, USA) equipped with a 5 mm inverse probe. The water resonance was suppressed using a NOESY presaturation pulse sequence. A total of 128 transients were acquired after eight steady-state scans. The interscan relaxation delay was set to 10 s. Before acquisition, each sample was shimmed to a TMSP linewidth less than 1 Hz. The resulting NMR spectra were processed and prepared for statistical data analysis using MetaboLabPy (version 0.9.29, https://pypi.org/project/metabolabpy/). Each free induction decay was exponentially line-broadened by 0.3 Hz and zero-filled to 131,072 data points before Fourier transformation. All spectra were then manually phase corrected. To prepare the NMR spectra for statistical data analysis, the left and right edges and the water region were excluded. A total of 32 areas were segmentally aligned. Noise filtering was applied to exclude data points below five times the standard deviation of the spectral noise.

The Chenomx software (Chenomx Inc., version 8) was used to determine metabolite concentrations. The metabolite concentration matrix from Chenomx was auto scaled before 27 specific metabolites were selected based on metabolic pathways that could be affected by CT inclusion in the diet. These selected metabolite concentrations underwent multivariate analysis using PCA [53] and partial least squares discriminant analysis (PLSDA) [65] was applied to the selected metabolites using the mixOmics package (version 6.28.0) [66]. Univariate analysis of rumen metabolite concentrations were completed using ANOVA [67] and Kruskal Wallis [68] based on distribution and homogeneity. Hierarchical cluster analysis (HCA) [69] between feed treatments using average linkage and the Euclidean distance metric were visualised using the pheatmap package (version 1.0.12) [70]. Annotation of the class of metabolite (e.g. protein, carbohydrate and fatty acid digestion) based its involvement in digestive pathways was adapted from the Kyoto encyclopaedia of genes and genomes (KEGG) database (Supplementary Table S9).

### 5.10 Analysis of correlations between fermentation and microbiome

Spearman’s rank correlation analysis was used between fermentation and microbiome data using the vegan package. Visualisation of correlations was performed using the corrplot package (version 0.94) [71].

## Supplementary Information

Supplementary material 1

## Data Availability

The datasets used and analysed during the current study are available alongside the R code used to perform the analysis on GitHub (https://github.com/TheHuwsLab/Willow_16s_analysis). The 16S RNA HiFi reads were deposited in ENA (https://www.ebi.ac.uk/ena/, uploaded on 26th January 2024) under the accession code ERS17888369/ERA28151579/. All other data is provided within the supplementary information files.

## Abbreviations

ADF: Acid detergent fibre
ADFI: Acid detergent fibre intake
ANOVA: Analysis of variance
AOAC: Association of official analytical chemists
ASV: Amplicon sequence variant
BG: Beagle feed treatment
BW: Body weight
CCS: Circular consensus sequencing
CDMI: Concentrate dry matter intake
CP: Crude protein
CRFI: Controlling and recording feed intake
CTs: Condensed tannins
CT: Condensed tannin
CTI: Condensed tannin inclusion
CTIntake: Daily Condensed tannin intake
DESeq2: Differential gene expression analysis
DLWG: Daily live weight gain
DM: Dry matter
DMI: Dry matter intake
DMSO: Dimethyl sulphoxide
DNA: Deoxyribose nucleic acid
EE: Ether extract
ENA: European nucleotide archive
FDMI: Forage dry matter intake
GCTU: Genomics core technology unit
GE: Gross energy
GEI: Gross energy intake
GF: GreenFeed
GS: Grass silage
HBAI: HCl-butanol-acetone-iron
HCA: Hierarchical cluster analysis
HP: Heat production
HPLC: High performance liquid chromatography
HSQC: Heteronuclear single quantum coherence
KEGG: Kyoto encyclopaedia of genes and genomes
LC-MS: Liquid chromatography mass spectrometer
LDA: Linear discriminant analysis
LRT: Likelihood ratio test
LW: Liveweight
ME: Metabolisable energy
MEI: Metabolisable energy intake
mDP: Mean degree of polymerisation
MJ: Mega joules
MS: Maize silage
NDF: Neutral detergent fibre
NDFI: Neutral detergent fibre intake
NI: Nitrogen intake
NIRS: Near infrared spectroscopy
NMR: Nuclear magnetic resonance
NOESY: Nuclear overhauser effect spectroscopy
NUE: Nitrogen use efficiency
OM: Organic matter
OMI: Organic matter intake
PC: Procyanidin
PCA: Principal component analysis
PD: Prodelphinidin
PERMANOVA: Permutational multivariate analysis of variance
PLSDA: Partial least squares discriminant analysis
QIIME: Quantitative insights into microbiology
RF: Rumen fluid
RNA: Ribonucleic acid
RQ: Respiratory quotient
s.e.m.: Standard error of the mean
SIL: Silage feed treatment
SMRT: Single molecule, real time
TDMI: Total dry matter intake
TMSP: Trimethylsilylpropanoic acid
TN: Terra Nova feed treatment
TSS: Total sum scaling
VFA: Volatile fatty acid
VST: Variance stabilising transformation
WI: Willow inclusion
WSC: Water soluble carbohydrate
