# Supplementary material for: Unlocking the potential of willow condensed tannins: effects on rumen fermentation, microbiome, and metabolome for sustainable ruminant nutrition
Source: Anim Microbiome. 2025 Jul 25;7:81. doi: 10.1186/s42523-025-00444-6 (PMC12297716; doi:10.1186/s42523-025-00444-6)
Supplement: Supplementary file 1 — Supplementary material 1 [file 42523_2025_444_MOESM1_ESM.docx]

**Supplementary material for**

# Unlocking the Potential of Willow Condensed Tannins: Effects on Rumen Fermentation, Microbiome, and Metabolome for Sustainable Ruminant Nutrition

Joshua P.Thompson, Omar Cristobal-Carballo, Tianhai Yan, Katie Lawther, Nicholas J. Dimonaco, Wayne E. Zeller, Zhenbin Zhang, Sharon Huws, Laudina Safo, Andrew D. Southam, Christian Ludwig, Gavin R. Lloyd, Sokratis Stergiadis, Katerina Theodoridou

**Table of contents**

**Supplementary Table S1.** Nutritional composition of each dietary component in the study 61

**Supplementary Table S2.** Nutritional composition of formulated treatment feeds 61

**Supplementary Figure S1.** Read counts per feed treatment 62

**Supplementary Table S3.** Alpha diversity indices for each feed treatment 62

**Supplementary Table S4.** Univariate analysis of relative abundance (%) from all feed treatments at Phylum level 63

**Supplementary Figure S2.** Relative abundance of ASV at Phylum level in rumen samples 64

**Supplementary Table S5.** Univariate analysis of relative abundance (%) from all feed treatments at Family level 65

**Supplementary Figure S3.** Relative abundance of ASV at Family level in rumen samples 66

**Supplementary Table S6.** Relative abundance (%) of shared genera between all feed treatments in descending order 67

**Supplementary Table S7.** Relative abundance (%) of specific genera unique to each feed treatment 71

**Supplementary Table S8.** Univariate analysis of selected Rumen metabolomics concentration (mmol/mL) from all feed treatments 73

**Supplementary Table S9.** Metabolic pathways and main metabolic classification of Rumen metabolites examined in this study 74

Supplementary Table S1. Nutritional composition of each dietary component in the study

|  | Nutritional Composition | | | |
| --- | --- | --- | --- | --- |
|  | SIL | BG | TN | C |
| DM (g/kg fresh) | 259.61 | 326.87 | 267.73 | 883.29 |
| Ash (g/kg DM) | 89.94 | 71.56 | 80.86 | 96.70 |
| OM (g/kg DM) | 910.06 | 928.44 | 919.14 | 903.30 |
| ADF (g/kg DM) | 347.87 | 303.82 | 391.82 | 152.53 |
| NDF (g/kg DM) | 543.74 | 385.69 | 509.01 | 245.14 |
| EE (g/kg DM) | 43.56 | 21.21 | 37.68 | 4040 |
| N (g/kg DM) | 24.77 | 34.61 | 37.71 | 27.92 |
| CP (g/kg DM) | 154.83 | 216.30 | 235.67 | 174.48 |
| GE (MJ/kg DM) | 19.50 | 20.16 | 20.18 | 17.72 |
| ME (MJ/kg DM) | 10.78 | 10.348 | 12.44 | 13.00 |
| CT (g/kgDM) | nd | 63.80 | 4.10 | nd |
| SIL, Silage control; BG, Salix. Beagle; TN, Salix Terra Nova; C, concentrate; DM, dry matter; OM, organic matter; ADF, acid detergent fibre; NDF, neutral detergent fibre; EE, ether extract; N, nitrogen; CP, crude protein; GE, gross energy; ME, metabolisable energy; CT, condensed tannin; nd, none detected | | | | |

Supplementary Table S2. Nutritional composition of formulated treatment feeds

|  | **Feed treatment diets** | | |
| --- | --- | --- | --- |
|  | **SIL** | **BG** | **TN** |
| DM (g/kg fresh) | 259.61 | 273.12 | 261.16 |
| Ash (g/kg DM) | 89.94 | 86.25 | 88.20 |
| OM (g/kg DM) | 910.06 | 913.75 | 911.80 |
| ADF (g/kg DM) | 347.87 | 339.02 | 356.269 |
| NDF (g/kg DM) | 543.74 | 511.99 | 537.10 |
| EE (g/kg DM) | 43.56 | 39.07 | 42.44 |
| N (g/kg DM) | 24.77 | 26.75 | 27.25 |
| CP (g/kg DM) | 154.83 | 167.18 | 170.28 |
| GE (MJ/kg DM) | 19.50 | 19.63 | 19.63 |
| ME (MJ/kg DM) | 10.78 | 10.69 | 11.09 |
| CT (g/kg DM) | Nd | 12.82 | 0.78 |
| CT (% DM) | Nd | 1.28 | 0.08 |
|  |  |  |  |
| SIL, Silage control; BG, Salix. Beagle; TN, Salix Terra Nova; DM, dry matter; OM, organic matter; ADF, acid detergent fibre; NDF, neutral detergent fibre; EE, ether extract; N, nitrogen; CP, crude protein; GE, gross energy; ME, metabolisable energy; CT, condensed tannin; Nd, none detected. | | | |


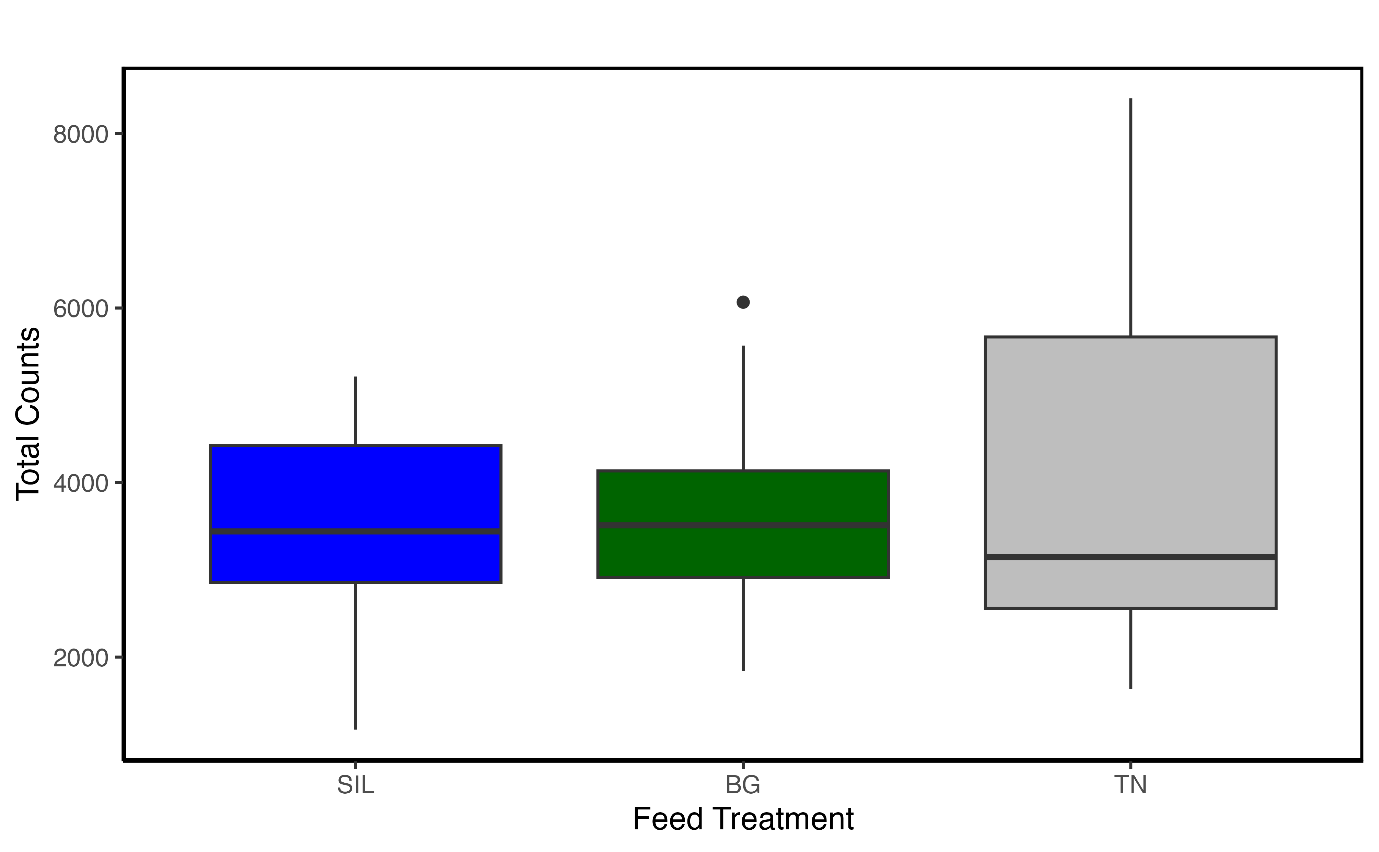


**Supplementary Figure S1. Quality control filtered and assigned with taxa read counts per feed treatment.** QIIME2 output read counts (Taxonomically classified - ASV). The central line in each box represents the median read count, while the lower and upper edges of the box indicate the first and third quartiles, respectively. Whiskers extend from the box to show the range of the data, excluding outliers, which are represented as individual points outside the whiskers. This graph provides insights into the variability and central tendency of sequencing depth across the sampled dataset.

Supplementary Table S3. Alpha diversity indices for each feed treatment

|  |  | **Treatment** | | |  |  |
| --- | --- | --- | --- | --- | --- | --- |
| Row |  | **SIL** | **BG** | **TN** | **s.e.m.** | **P-value** |
| 1 | Chao1 Richness | 125 | 137 | 136 | 5.33 | 0.520 |
| 2 | Pielou’s Evenness | 0.794 | 0.798 | 0.804 | 0.00496 | 0.724 |
| 3 | Inverse Simpson | 23.9 | 26.8 | 27.3 | 1.05 | 0.361 |

**Supplementary Table S4. Univariate analysis of relative abundance (%) from all feed treatments at Phylum level.** Analysis of Variance (ANOVA) and Kruskal Wallis test was performed on phylum measuring the impact of feed treatment on phylum relative abundance (%). The code ‘***’, denotes level of significance P<0.001 in differences amongst feed treatment.

|  |  | **Treatment** | | |  |  |
| --- | --- | --- | --- | --- | --- | --- |
| **Row** | **Phylum** | **SIL** | **BG** | **TN** | **s.e.m.** | **P-value** |
| 1 | *Bacteroidota* | 40.6 | 42.8 | 41.1 | 0.775 | 0.429 |
| 2 | *Firmicutes_A* | 21.4 | 22.8 | 21.5 | 0.678 | 0.520 |
| 3 | *Verrucomicrobiota* | 10.6 | 9.78 | 10.9 | 0.759 | 0.916 |
| 4 | *Planctomycetota* | 9.42 | 8.00 | 8.76 | 0.754 | 0.300 |
| 5 | *Firmicutes_D* | 7.52 | 6.79 | 6.86 | 0.392 | 0.965 |
| 6 | *Patescibacteria* | 2.51 | 2.23 | 2.73 | 0.217 | 0.479 |
| 7 | *Firmicutes_C* | 1.71 | 1.33 | 1.31 | 0.107 | 0.237 |
| 8 | *Fibrobacterota* | 1.41 | 0.0962 | 0.217 | 0.242 | 0.320 |
| 9 | *Proteobacteria* | 1.24 | 1.55 | 1.25 | 0.231 | 0.560 |
| 10 | *Actinobacteriota* | 0.944^a^ | 2.22^b^ | 2.82^b^ | 0.239 | *** |
| 11 | *Unknown_d__Bacteria* | 0.523 | 0.567 | 0.417 | 0.132 | 0.247 |
| 12 | *Spirochaetota* | 0.496 | 0.395 | 0.575 | 0.0628 | 0.160 |
| 13 | *Cyanobacteria* | 0.438 | 0.227 | 0.199 | 0.0601 | 0.0921 |
| 14 | *Chloroflexota* | 0.404 | 0.162 | 0.286 | 0.0445 | 0.210 |
| 15 | *Methanobacteriota_A_1229* | 0.319 | 0.387 | 0.409 | 0.0340 | 0.541 |
| 16 | *Eremiobacterota* | 0.176 | 0.0806 | 0.0962 | 0.0343 | 0.723 |
| 17 | *Armatimonadota* | 0.0580 | 0.0687 | 0.0583 | 0.0160 | 0.938 |
| 18 | *Desulfobacterota_I* | 0.0457 | 0.254 | 0.314 | 0.0465 | 0.411 |
| 19 | *Campylobacterota* | 0.0376 | 0.0311 | 0.00198 | 0.00972 | 0.304 |
| 20 | *Riflebacteria* | 0.0357 | 0.0428 | 0.0111 | 0.0103 | 0.483 |
| 21 | *Firmicutes_B_370539* | 0.0333 | 0.0913 | 0.0789 | 0.0169 | 0.210 |
| 22 | *Elusimicrobiota* | 0.0237 | 0 | 0 | 0.00689 | 0.128 |
| 23 | *Fusobacteriota* | 0.0121 | 0 | 0.0222 | 0.00564 | 0.169 |
| 24 | *Desulfobacterota_G_459546* | 0.00690 | 0.0240 | 0.0140 | 0.00727 | 0.797 |
| 25 | *Bdellovibrionota_E* | 0 | 0.00480 | 0 | 0.00160 | 0.368 |
| 26 | *Synergistota* | 0 | 0.00961 | 0 | 0.00320 | 0.368 |
| 27 |  |  |  |  |  |  |
| 28 | *Firmicutes:Bacteroidota* | 0.786 | 0.729 | 0.726 | 0.0293 | 0.895 |
|  |  |  |  |  |  |  |
| SIL, silage; BG, Beagle; TN, Terra Nova; s.e.m, standard error of the mean | | | | | | |


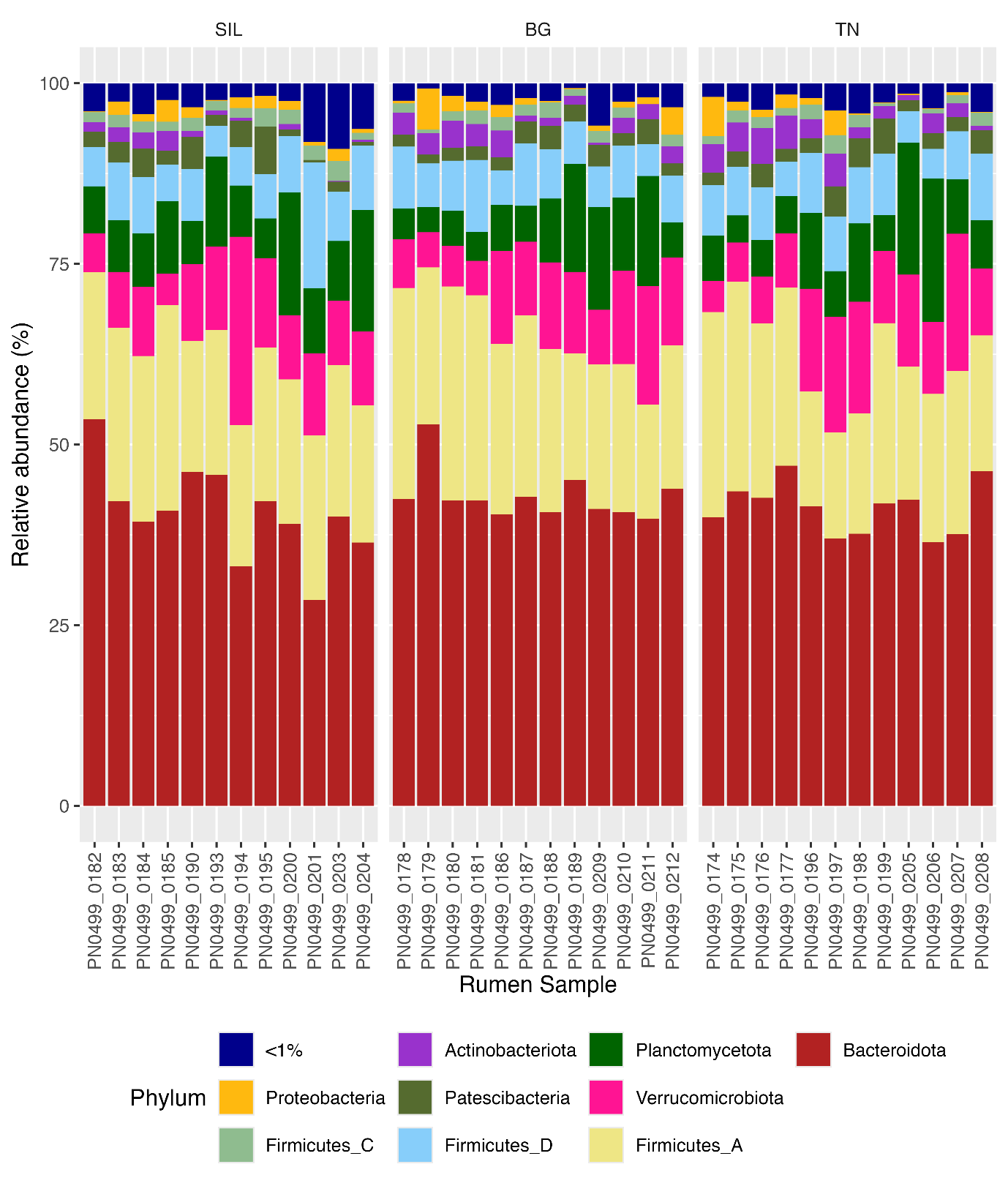


**Supplementary Figure S2.** **Relative abundance of ASV at Phylum level in rumen samples.** Stacked bar charts illustrate the average relative abundances in percentage, which were calculated by total sum scaling of ASV read counts classified at phylum level. Taxonomic groups that constituted less than 1% of the total abundance are aggregated into a category labelled ‘<1%'. Samples were indicated on the X-axis while relative abundance (%) indicated on the y-axis. Each colour in the chart corresponds to a different predominant phylum, as indicated in the legend. Each facet is according to Feed treatment.

**Supplementary Table S5. Univariate analysis of relative abundance (%) from all feed treatments at Family level with any families with an abundance of <1% combined as the <1% abundant family.** Analysis of Variance (ANOVA) and Kruskal Wallis test was performed on phylum measuring the impact of feed treatment on phylum relative abundance (%). The code ‘*’, ‘**’ and ‘***’ denotes level of significance P<0.05, P<0.01 and P<0.001 respectively in differences amongst feed treatment.

|  |  | Treatment | | |  |  |
| --- | --- | --- | --- | --- | --- | --- |
| Row | Family | SIL | BG | TN | s.e.m. | P-value |
| 1 | *Bacteroidaceae* | 26.9 | 25.9 | 23.3 | 0.772 | 0.0546 |
| 2 | *Thermoguttaceae* | 9.37 | 7.99 | 8.71 | 0.754 | 0.292 |
| 3 | *Lachnospiraceae* | 7.65 | 9.85 | 8.79 | 0.511 | 0.235 |
| 4 | *UBA932* | 5.15^a^ | 6.84^b^ | 6.55^b^ | 0.258 | * |
| 5 | *CAG-74* | 5.05 | 3.88 | 3.69 | 0.332 | 0.258 |
| 6 | *UBA1067* | 4.92 | 3.92 | 4.47 | 0.265 | 0.252 |
| 7 | *UBA660* | 3.99 | 5.22 | 5.24 | 0.307 | 0.132 |
| 8 | *F082* | 3.10^a^ | 4.01^ab^ | 5.57^b^ | 0.348 | * |
| 9 | *Anaeroplasmataceae* | 2.66 | 0.54 | 0.41 | 0.443 | 0.773 |
| 10 | *Nanosyncoccaceae* | 2.51 | 2.19 | 2.68 | 0.220 | 0.643 |
| 11 | *Anaerovoracaceae* | 2.49 | 2.36 | 2.50 | 0.159 | 0.922 |
| 12 | *Lenti-01* | 2.35 | 1.80 | 3.06 | 0.605 | 0.430 |
| 13 | *P3* | 1.80 | 1.73 | 1.55 | 0.185 | 0.495 |
| 14 | *UBA3636* | 1.77^a^ | 3.04^b^ | 2.70^b^ | 0.181 | ** |
| 15 | *Acutalibacteraceae* | 1.62 | 1.40 | 1.71 | 0.126 | 0.569 |
| 16 | *Acidaminococcaceae* | 1.56 | 1.27 | 1.19 | 0.105 | 0.340 |
| 17 | *WCHB1-69* | 1.23 | 1.27 | 1.27 | 0.115 | 0.761 |
| 18 | *Oscillospiraceae_88309* | 1.18^a^ | 2.29^b^ | 1.51^a^ | 0.133 | *** |
| 19 | *Muribaculaceae* | 0.973 | 1.63 | 1.55 | 0.158 | 0.180 |
| 20 | *Atopobiaceae* | 0.719^a^ | 2.02^b^ | 2.61^b^ | 0.234 | ** |
| 21 | <1% | 0.116 | 0.0971 | 0.0972 | 0.00499 | 0.549 |
|  |  |  |  |  |  |  |
| SIL, silage; BG, Beagle; TN, Terra Nova; s.e.m, standard error of Mean; <1%, families with abundance <1% are combined to give a <1% abundant family. | | | | | | |


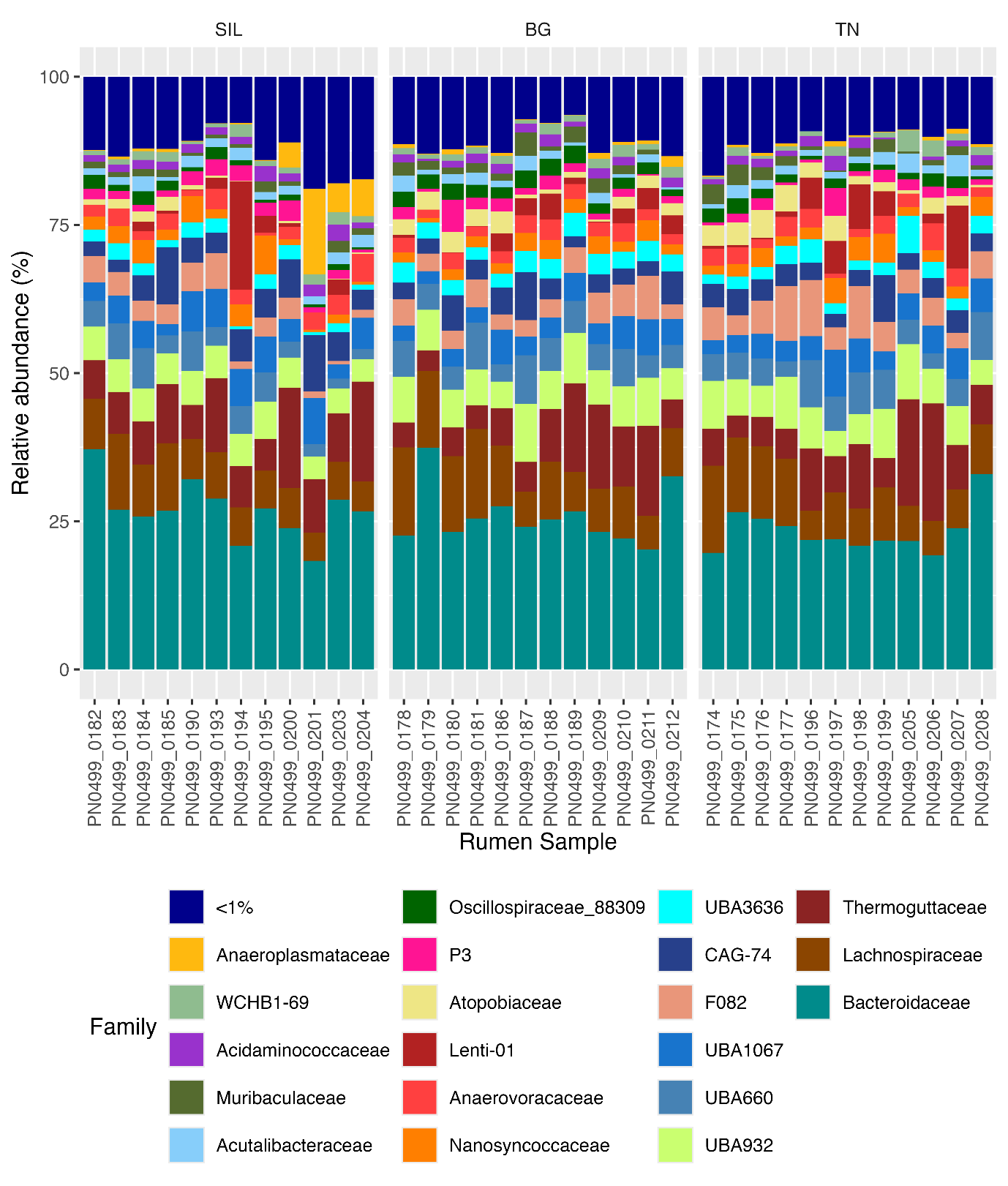


**Supplementary Figure S3. Relative abundance of ASV at Family level in rumen samples.** Stacked bar charts illustrate the average relative abundances in percentage, which were calculated by total sum scaling of ASV read counts classified at family level. Taxonomic groups that constituted less than 1% of the total abundance are aggregated into a category labelled ‘<1%'. Samples were indicated on the X-axis while relative abundance (%) indicated on the y-axis. Each colour in the chart corresponds to a different predominant family, as indicated in the legend. Each facet is according to Feed treatment.

**Supplementary Table S6. Relative abundance (%) of shared genera between all feed treatments in descending order**

|  | Treatment Relative Abundance (%) | | |
| --- | --- | --- | --- |
| Genus | SIL | BG | TN |
| *Prevotella* | 20.346 | 20.373 | 17.588 |
| *DSXL01* | 9.373 | 7.992 | 8.714 |
| *unknown_f__Lachnospiraceae* | 5.671 | 7.144 | 6.239 |
| *Cryptobacteroides* | 3.664 | 5.306 | 5.061 |
| *Limimorpha* | 3.102 | 4.011 | 5.573 |
| *UBA4334* | 4.656 | 3.239 | 3.517 |
| *SFMI01* | 4.569 | 3.519 | 3.224 |
| *UBA1067* | 3.438 | 2.221 | 2.762 |
| *UBA3636* | 1.771 | 3.037 | 2.702 |
| *Lenti-01* | 2.346 | 1.801 | 3.063 |
| *UBA1711* | 1.752 | 1.729 | 1.553 |
| *DUPH01* | 1.454 | 1.692 | 1.697 |
| *Nanosyncoccus* | 1.681 | 1.386 | 1.706 |
| *Paraprevotella* | 1.424 | 1.504 | 1.561 |
| *Succiniclasticum* | 1.561 | 1.273 | 1.188 |
| *Mogibacterium* | 1.411 | 1.181 | 1.159 |
| *Sodaliphilus* | 0.433 | 1.570 | 1.450 |
| *Limivicinus* | 0.886 | 1.244 | 1.293 |
| *unknown_f__UBA660* | 0.927 | 1.508 | 0.943 |
| *Saccharofermentans* | 1.120 | 0.844 | 1.025 |
| *F23-D06* | 0.968 | 1.080 | 0.931 |
| *unknown_f__Atopobiaceae* | 0.355 | 1.034 | 1.298 |
| *UBA2834* | 0.824 | 0.806 | 0.970 |
| *Egerieousia* | 0.796 | 0.924 | 0.812 |
| *Oribacterium* | 0.801 | 0.923 | 0.798 |
| *UBA3789* | 0.688 | 0.863 | 0.778 |
| *Ruminococcus_E* | 0.746 | 0.626 | 0.907 |
| *Copromorpha* | 0.658 | 0.751 | 0.844 |
| *Anaeroplasma* | 1.726 | 0.241 | 0.209 |
| *Parafannyhessea* | 0.190 | 0.735 | 1.120 |
| *Bact-11* | 0.687 | 0.613 | 0.675 |
| *unknown_f__Acutalibacteraceae* | 0.668 | 0.598 | 0.700 |
| *RF16* | 0.776 | 0.601 | 0.494 |
| *Fibrobacter* | 1.411 | 0.096 | 0.217 |
| *Ruminococcus_D* | 0.790 | 0.334 | 0.430 |
| *CAG-605* | 0.482 | 0.440 | 0.614 |
| *unknown_d__Bacteria* | 0.523 | 0.567 | 0.415 |
| *RZZM01* | 0.665 | 0.550 | 0.268 |
| *Treponema_D* | 0.469 | 0.372 | 0.520 |
| *Butyrivibrio_A_168226* | 0.226 | 0.644 | 0.484 |
| *unknown_f__Anaeroplasmataceae* | 0.870 | 0.269 | 0.174 |
| *UBA6985* | 0.342 | 0.483 | 0.470 |
| *unknown_f__CAG-74* | 0.455 | 0.335 | 0.417 |
| *RUG12783* | 0.334 | 0.361 | 0.508 |
| *Faecousia* | 0.162 | 0.779 | 0.121 |
| *UBA1179* | 0.311 | 0.332 | 0.367 |
| *G11* | 0.315 | 0.286 | 0.390 |
| *UBA1732* | 0.556 | 0.153 | 0.197 |
| *unknown_f__CAG-508* | 0.293 | 0.242 | 0.347 |
| *Flexilinea* | 0.401 | 0.162 | 0.286 |
| *RUG705* | 0.170 | 0.288 | 0.380 |
| *unknown_f__Anaerovoracaceae* | 0.301 | 0.235 | 0.285 |
| *Alysiella* | 0.116 | 0.370 | 0.334 |
| *unknown_f__Bacteroidaceae* | 0.143 | 0.367 | 0.288 |
| *UMGS1994* | 0.188 | 0.297 | 0.301 |
| *OLB9* | 0.291 | 0.059 | 0.326 |
| *CAG-1000* | 0.158 | 0.149 | 0.340 |
| *Ruminococcus_C_59129* | 0.235 | 0.233 | 0.176 |
| *RUG11690* | 0.227 | 0.113 | 0.284 |
| *UBA1367* | 0.168 | 0.245 | 0.191 |
| *Desulfovibrio_R_446353* | 0.042 | 0.243 | 0.308 |
| *RUG13038* | 0.141 | 0.239 | 0.210 |
| *Pseudobutyrivibrio* | 0.086 | 0.121 | 0.367 |
| *unknown_f__Eggerthellaceae* | 0.198 | 0.166 | 0.190 |
| *UBA1407* | 0.179 | 0.252 | 0.120 |
| *Duodenibacillus* | 0.184 | 0.199 | 0.119 |
| *unknown_o__Bacteroidales* | 0.198 | 0.131 | 0.156 |
| *CAG-269* | 0.085 | 0.207 | 0.178 |
| *RUG12438* | 0.192 | 0.170 | 0.104 |
| *unknown_f__Coprobacillaceae* | 0.113 | 0.147 | 0.195 |
| *RUG14515* | 0.116 | 0.148 | 0.162 |
| *Methanobrevibacter_A* | 0.097 | 0.161 | 0.157 |
| *unknown_f__Gastranaerophilaceae* | 0.242 | 0.123 | 0.046 |
| *Bulleidia* | 0.083 | 0.114 | 0.211 |
| *Methanobrevibacter_D_1148* | 0.123 | 0.163 | 0.119 |
| *RUG472* | 0.112 | 0.135 | 0.156 |
| *CAG-873* | 0.355 | 0.013 | 0.029 |
| *unknown_c__Gammaproteobacteria* | 0.224 | 0.081 | 0.078 |
| *Eubacterium_Q* | 0.150 | 0.140 | 0.093 |
| *unknown_f__Oscillospiraceae_88309* | 0.094 | 0.248 | 0.040 |
| *Porcincola* | 0.102 | 0.147 | 0.108 |
| *RUG521* | 0.096 | 0.146 | 0.105 |
| *CAG-710* | 0.079 | 0.138 | 0.128 |
| *UBA1394* | 0.167 | 0.113 | 0.055 |
| *Avispirillum* | 0.066 | 0.159 | 0.108 |
| *RUG410* | 0.091 | 0.092 | 0.144 |
| *UBA3830* | 0.081 | 0.072 | 0.164 |
| *Faecimonas* | 0.156 | 0.083 | 0.071 |
| *UBA2813* | 0.149 | 0.051 | 0.105 |
| *unknown_f__Muribaculaceae* | 0.185 | 0.046 | 0.073 |
| *unknown_f__Neisseriaceae_563222* | 0.057 | 0.164 | 0.081 |
| *RUG11194* | 0.091 | 0.047 | 0.160 |
| *CAG-914* | 0.004 | 0.101 | 0.190 |
| *Streptococcus* | 0.022 | 0.094 | 0.171 |
| *unknown_f__Selenomonadaceae_42771* | 0.143 | 0.035 | 0.095 |
| *UMGS1449* | 0.091 | 0.109 | 0.072 |
| *RUG13615* | 0.061 | 0.106 | 0.067 |
| *Coprosoma* | 0.038 | 0.071 | 0.117 |
| *unknown_c__Xenobia* | 0.138 | 0.028 | 0.057 |
| *Anaerobutyricum* | 0.053 | 0.124 | 0.045 |
| *unknown_f__Dehalobacteriaceae* | 0.033 | 0.091 | 0.074 |
| *unknown_c__Bacteroidia* | 0.079 | 0.016 | 0.100 |
| *DSUL01* | 0.058 | 0.069 | 0.058 |
| *PeH17* | 0.046 | 0.045 | 0.088 |
| *Methanobacterium_D_900* | 0.058 | 0.052 | 0.066 |
| *UBA2253* | 0.056 | 0.066 | 0.047 |
| *Catonella* | 0.007 | 0.084 | 0.077 |
| *Moraxella_C_651924* | 0.029 | 0.046 | 0.088 |
| *CAG-417* | 0.081 | 0.029 | 0.046 |
| *C-53* | 0.061 | 0.047 | 0.042 |
| *CAG-177* | 0.025 | 0.093 | 0.026 |
| *CAG-302* | 0.014 | 0.006 | 0.119 |
| *CAG-273* | 0.064 | 0.041 | 0.033 |
| *Eubacterium_R* | 0.099 | 0.028 | 0.010 |
| *RUG11420* | 0.038 | 0.042 | 0.052 |
| *unknown_f__Marinifilaceae* | 0.010 | 0.059 | 0.062 |
| *Bibersteinia* | 0.003 | 0.085 | 0.042 |
| *Limenecus* | 0.039 | 0.039 | 0.046 |
| *UBA3206* | 0.065 | 0.033 | 0.023 |
| *Scybalousia* | 0.023 | 0.059 | 0.037 |
| *unknown_f__Pasteurellaceae* | 0.044 | 0.060 | 0.014 |
| *UBA4951* | 0.036 | 0.043 | 0.038 |
| *unknown_f__Methanobacteriaceae* | 0.041 | 0.010 | 0.064 |
| *Eubacterium_T* | 0.035 | 0.012 | 0.066 |
| *unknown_f__DSM-8532* | 0.008 | 0.062 | 0.036 |
| *SZUA-421* | 0.049 | 0.010 | 0.045 |
| *RUG13077* | 0.036 | 0.036 | 0.031 |
| *UBA3207* | 0.051 | 0.020 | 0.033 |
| *Onthenecus* | 0.021 | 0.029 | 0.049 |
| *UBA1205* | 0.007 | 0.058 | 0.034 |
| *unknown_f__Ruminococcaceae* | 0.037 | 0.031 | 0.029 |
| *UBA2450* | 0.069 | 0.013 | 0.014 |
| *Onthocola_B* | 0.019 | 0.027 | 0.049 |
| *Mannheimia* | 0.005 | 0.029 | 0.061 |
| *Shuttleworthia* | 0.029 | 0.049 | 0.017 |
| *unknown_c__Alphaproteobacteria* | 0.037 | 0.019 | 0.038 |
| *Ruminococcus_C_58660* | 0.022 | 0.041 | 0.027 |
| *Rifleibacterium* | 0.036 | 0.043 | 0.011 |
| *CAG-533* | 0.006 | 0.073 | 0.007 |
| *Victivallis* | 0.020 | 0.028 | 0.037 |
| *Fimenecus* | 0.019 | 0.039 | 0.026 |
| *Hornefia* | 0.025 | 0.012 | 0.044 |
| *Malacoplasma_A_271179* | 0.028 | 0.018 | 0.034 |
| *Porphyromonas_A_859423* | 0.011 | 0.014 | 0.053 |
| *Eubacterium_G* | 0.015 | 0.037 | 0.024 |
| *unknown_c__Spirochaetia* | 0.021 | 0.018 | 0.035 |
| *Bact-19* | 0.047 | 0.016 | 0.009 |
| *unknown_f__Weeksellaceae* | 0.009 | 0.042 | 0.021 |
| *Campylobacter_B* | 0.038 | 0.031 | 0.002 |
| *HUN007* | 0.009 | 0.013 | 0.046 |
| *Xenobium* | 0.016 | 0.037 | 0.014 |
| *unknown_o__Lachnospirales* | 0.015 | 0.036 | 0.015 |
| *Ruminiclostridium_E* | 0.018 | 0.027 | 0.021 |
| *unknown_f__CAG-138* | 0.013 | 0.034 | 0.018 |
| *NK4A144* | 0.038 | 0.009 | 0.018 |
| *UBA5026* | 0.009 | 0.015 | 0.040 |
| *Enteromonas* | 0.033 | 0.015 | 0.014 |
| *Bruticola* | 0.022 | 0.016 | 0.024 |
| *UBA1195* | 0.022 | 0.019 | 0.018 |
| *UBA6448* | 0.033 | 0.012 | 0.013 |
| *UBA6857* | 0.014 | 0.010 | 0.032 |
| *UBA636* | 0.023 | 0.019 | 0.011 |
| *unknown_o__Acholeplasmatales* | 0.022 | 0.023 | 0.007 |
| *UBA4292* | 0.006 | 0.021 | 0.022 |
| *unknown_f__UBA1067* | 0.032 | 0.009 | 0.006 |
| *unknown_o__Desulfuromonadales* | 0.007 | 0.024 | 0.014 |
| *WRMH01* | 0.008 | 0.006 | 0.029 |
| *JAACSU01* | 0.003 | 0.017 | 0.016 |
| *Selenomonas_B_42753* | 0.010 | 0.013 | 0.011 |
| *Bilifractor* | 0.010 | 0.010 | 0.014 |
| *UBA1248* | 0.008 | 0.009 | 0.016 |
| *unknown_c__Kiritimatiellae_777934* | 0.017 | 0.012 | 0.002 |
| *unknown_f__Christensenellaceae* | 0.015 | 0.004 | 0.011 |
| *CAG-274* | 0.010 | 0.005 | 0.013 |
| *SFLA01* | 0.003 | 0.012 | 0.010 |
| *Onthomonas* | 0.005 | 0.004 | 0.015 |
| *XBB2008* | 0.009 | 0.003 | 0.010 |
| *UBA644* | 0.005 | 0.005 | 0.012 |
| *UBA9732* | 0.005 | 0.005 | 0.011 |
| *Zag111* | 0.008 | 0.009 | 0.003 |
| *Neisseria_563205* | 0.007 | 0.008 | 0.004 |
| *RUG721* | 0.005 | 0.005 | 0.003 |

**Supplementary Table S7. Relative abundance (%) of specific genera unique to each feed treatment**

|  |  | Relative Abundance (%) | | |
| --- | --- | --- | --- | --- |
| Row | Genus | SIL | BG | TN |
| 1 | *GN02-873* | 0 | 0 | 0.00793 |
| 2 | *Kingella_B_563181* | 0 | 0 | 0.00898 |
| 3 | *Caviibacter* | 0 | 0 | 0.0098 |
| 4 | *GWC1-27-15* | 0 | 0 | 0.00838 |
| 5 | *Soleaferrea* | 0 | 0 | 0.00838 |
| 6 | *Actinobacillus_C_733309* | 0 | 0 | 0.00496 |
| 7 | *GWA2-37-10* | 0 | 0 | 0.00496 |
| 8 | *unknown_o__Coriobacteriales* | 0 | 0 | 0.00699 |
| 9 | *UBA1402* | 0 | 0 | 0.00748 |
| 10 | *Blautia_A_141781* | 0 | 0 | 0.00748 |
| 11 | *UBA2177* | 0 | 0 | 0.0138 |
| 12 | *Porphyromonas_A_859426* | 0 | 0 | 0.00397 |
| 13 | *WRJS01* | 0 | 0 | 0.00455 |
| 14 | *BICA1-8* | 0 | 0 | 0.00449 |
| 15 | *Anaerofustis* | 0 | 0 | 0.00974 |
| 16 | *Christensenella* | 0 | 0 | 0.00341 |
| 17 | *UMGS2069* | 0 | 0 | 0.00765 |
| 18 | *Propionibacterium* | 0 | 0 | 0.00198 |
| 19 | *F0058* | 0 | 0 | 0.00198 |
| 20 | *unknown_f__CAG-826* | 0 | 0 | 0.00198 |
| 21 | *Peptostreptococcus* | 0 | 0 | 0.00299 |
| 22 | *Methanosphaera* | 0 | 0 | 0.00299 |
| 23 | *unknown_f__Nanoperiomorbaceae* | 0 | 0 | 0.00633 |
| 24 | *Scatocola* | 0 | 0.00961 | 0 |
| 25 | *Eubacterium_S* | 0 | 0.00442 | 0 |
| 26 | *Choladousia* | 0 | 0.00748 | 0 |
| 27 | *Fretibacterium* | 0 | 0.00687 | 0 |
| 28 | *Aphodomorpha* | 0 | 0.0125 | 0 |
| 29 | *unknown_o__Burkholderiales_592524* | 0 | 0.00599 | 0 |
| 30 | *Selenomonas_A* | 0 | 0.00662 | 0 |
| 31 | *Succinivibrio* | 0 | 0.00864 | 0 |
| 32 | *Onthovivens* | 0 | 0.00436 | 0 |
| 33 | *WM01* | 0 | 0.00329 | 0 |
| 34 | *unknown_c__Clostridia_258483* | 0 | 0.00905 | 0 |
| 35 | *unknown_f__CAG-552* | 0 | 0.00275 | 0 |
| 36 | *unknown_f__Dethiosulfovibrionaceae* | 0 | 0.00275 | 0 |
| 37 | *unknown_f__Actinomycetaceae* | 0 | 0.00571 | 0 |
| 38 | *CAG-196* | 0 | 0.00571 | 0 |
| 39 | *UBA1412* | 0.123 | 0 | 0 |
| 40 | *unknown_f__P3* | 0.0452 | 0 | 0 |
| 41 | *Ga6A1* | 0.0428 | 0 | 0 |
| 42 | *UBA1436* | 0.0237 | 0 | 0 |
| 43 | *SFDB01* | 0.00936 | 0 | 0 |
| 44 | *UBA1213* | 0.00777 | 0 | 0 |
| 45 | *WQSK01* | 0.00702 | 0 | 0 |
| 46 | *unknown_f__CAG-272* | 0.00702 | 0 | 0 |
| 47 | *CAG-41* | 0.00583 | 0 | 0 |
| 48 | *Limousia* | 0.00468 | 0 | 0 |
| 49 | *WRAI01* | 0.00389 | 0 | 0 |
| 50 | *WRNP01* | 0.0032 | 0 | 0 |
| 51 | *Cupidesulfovibrio* | 0.00393 | 0 | 0 |
| 52 | *CAG-1252* | 0.00393 | 0 | 0 |
| 53 | *unknown_f__CAG-239* | 0.00854 | 0 | 0 |

**Supplementary Table S8. Univariate analysis of selected rumen metabolomics concentration (mmol/mL) from all feed treatments.** Analysis of Variance (ANOVA) and Kruskal Wallis test was performed on selected rumen metabolomics measuring the impact of feed treatment.

|  |  | **Treatment** | | |  |  |
| --- | --- | --- | --- | --- | --- | --- |
| **Row** |  | **SIL** | **BG** | **TN** | **s.e.m.** | **P-value** |
| 1 | **Protein** |  |  |  |  |  |
| 2 | **3-Hydroxyphenyacetate** | 0.0272 | 0.0250 | 0.0267 | 0.00299 | 0.700 |
| 3 | **Alanine** | 0.0525 | 0.0548 | 0.0810 | 0.00621 | 0.129 |
| 4 | **Aspartate** | 0.0507 | 0.0644 | 0.0843 | 0.00688 | 0.0877 |
| 5 | **Cadaverine** | 0.00248 | 0.00395 | 0.00271 | 0.000480 | 0.370 |
| 6 | **Glutamate** | 0.167 | 0.215 | 0.234 | 0.0128 | 0.0611 |
| 7 | **Glycine** | 0.0307 | 0.0352 | 0.0511 | 0.00611 | 0.471 |
| 8 | **Histidine** | 0.00726 | 0.00631 | 0.00245 | 0.00124 | 0.490 |
| 9 | **Isobutyrate** | 0.546 | 0.646 | 0.675 | 0.0440 | 0.361 |
| 10 | **Isoleucine** | 0.0197 | 0.0311 | 0.0225 | 0.00319 | 0.648 |
| 11 | **Isovalerate** | 0.0614 | 0.0574 | 0.0670 | 0.00707 | 0.758 |
| 12 | **Leucine** | 0.0135 | 0.0335 | 0.0335 | 0.00428 | 0.115 |
| 13 | **Lysine** | 0.0151 | 0.0263 | 0.0278 | 0.00305 | 0.355 |
| 14 | **N-Phenylacetylglycine** | 0.0126 | 0.0128 | 0.0152 | 0.00106 | 0.545 |
| 15 | **Phenylacetate** | 0.0604 | 0.0853 | 0.108 | 0.0132 | 0.210 |
| 16 | **Proline** | 0.0938 | 0.102 | 0.125 | 0.00593 | 0.141 |
| 17 | **Tryosine** | 0.0315 | 0.0286 | 0.0356 | 0.00260 | 0.511 |
| 18 | **Carbohydrate** |  |  |  |  |  |
| 19 | **Acetate** | 55.0 | 47.3 | 43.7 | 3.38 | 0.159 |
| 20 | **Butyrate** | 4.33 | 4.19 | 3.87 | 0.269 | 0.583 |
| 21 | **Lactose** | 0.0992 | 0.0753 | 0.117 | 0.00803 | 0.0757 |
| 22 | **Maltose** | 0.0642 | 0.0519 | 0.0654 | 0.00459 | 0.200 |
| 23 | **Propionate** | 10.9 | 8.88 | 8.90 | 0.719 | 0.126 |
| 24 | **Organic Acid** |  |  |  |  |  |
| 25 | **3-Phenylpropionate** | 0.235 | 0.214 | 0.210 | 0.0181 | 0.820 |
| 26 | **Gentisate** | 0.00705 | 0.0175 | 0.0200 | 0.00297 | 0.242 |
| 27 | **Maleate** | 0.000930 | 0.000640 | 0.000740 | 0.0000900 | 0.606 |
| 28 | **Fatty Acid** |  |  |  |  |  |
| 29 | **Benzoate** | 0.0158 | 0.0163 | 0.0200 | 0.00109 | 0.198 |
| 30 | **Succinate** | 0.137 | 0.157 | 0.175 | 0.0191 | 0.550 |
| 31 | **Vitamin** |  |  |  |  |  |
| 32 | **Pantothenate** | 0.0114 | 0.0130 | 0.0100 | 0.00161 | 0.871 |
|  |  |  |  |  |  |  |
| SIL, silage; BG, Beagle; TN, Terra Nova | | | | | | |

**Supplementary Table S9. Metabolic pathways and main metabolic classification of Rumen metabolites examined in this study.**

| Row | Metabolite | Metabolism Pathways | Metabolic Classification |
| --- | --- | --- | --- |
| 1 | 3-Hydroxyphenylacetate | Tyrosine  Phenylalanine  Styrene | Protein |
| 2 | Alanine | Cyanoamino acid | Protein |
| 3 | Aspartate | Arginine biosynthesis  Alanine, aspartate and glutamate metabolism  Glycine, serine and threonine metabolism  Monobactam biosynthesis  Cysteine and methionine metabolism  Lysine biosynthesis  Histidine metabolism  beta-Alanine metabolism  Cyanoamino acid metabolism  D-Amino acid metabolism  Carbon fixation by Calvin cycle  Nicotinate and nicotinamide metabolism  Pantothenate and CoA biosynthesis  Aminoacyl-tRNA biosynthesis  Biosynthesis of various other secondary metabolites  Biosynthesis of various antibiotics  Biosynthesis of various plant secondary metabolites  Biosynthesis of plant secondary metabolites  Biosynthesis of alkaloids derived from ornithine, lysine and nicotinic acid  Biosynthesis of plant hormones  Metabolic pathways  Biosynthesis of secondary metabolites  Microbial metabolism in diverse environments  Carbon metabolism  2-Oxocarboxylic acid metabolism  Biosynthesis of amino acids  Biosynthesis of cofactors  ABC transporters  Two-component system  Bacterial chemotaxis  Neuroactive ligand-receptor interaction  Protein digestion and absorption  Central carbon metabolism in cancer | Protein |
| 4 | Cadaverine | Lysine degradation  D-amino acid  Glutathione  Tropane, piperidine and pyridine alkaloid biosynthesis  Biosynthesis of plant secondary metabolites7Biosynthesis of alkaloids derived from ornithine, lysine and nicotinic acid  Biosynthesis of secondary metabolites  Protein digestion and absorption | Protein |
| 5 | Glutamate | Cyanoamino acid | Protein |
| 6 | Glycine | Primary bile acid  Purine  Glycine, serine and threonine  Lysine  Phosphonate and phosphinate  Cyanoamino acid  Glutathione  Glyoxylate and dicarboxylate  One carbon pool by folate  Methane  Thiamine  Lipoic  Porphyrin  Aminoacyl –tRNA  Biosynthesis of plant secondary metabolites  Biosynthesis of secondary metabolites  Carbon  Biosynthesis of amino acids  Biosynthesis of cofactors  Vancomycin resistance  ABC transporters  Biofilm restoration – Escherichia coli  Neuroactive ligand – receptor interaction  Synaptic vesicle cycle  Protein digestion and absorption  Mineral absorption  Central carbon metabolism in cancer | Protein |
| 7 | Histidine | Histidine  Staurosporine biosynthesis  Beta-Alanine  D-amino acid  Aminoacyl – tRNA biosynthesis  Biosynthesis of plant secondary metabolites  Biosynthesis of alkaloids derived from histidine and purine  Biosynthesis of secondary metabolites  Biosynthesis of amino acids  ABC transporters  Protein digestion and absorption  Central carbon metabolism in cancer | Protein |
| 8 | Isobutyrate | Degradation of aromatic compounds  Protein digestion and absorption | Protein |
| 9 | Isoleucine | Valine, leucine and isoleucine degradation  Valine, leucine and isoleucine biosynthesis  Cyanoamino acid  Tropane, piperidine and pyridine alkaloid biosynthesis  Glucosinolate biosynthesis  Aminoacyl – tRNA biosynthesis  Biosynthesis of plant secondary metabolites  Biosynthesis of alkaloids derived from ornithine, lysine and nicotinic acids  Biosynthesis of secondary metabolites  2-Oxocarboxylic acid  Biosynthesis of amino acids  ABC transporters  Protein digestion and absorption  Mineral absorption  Shigellosis  Central carbon metabolism | Protein |
| 10 | Isovalerate | Biosynthesis of alkaloids derived from histidine and purine  Biosynthesis of secondary metabolites  Protein digestion and absorption | Protein |
| 11 | Leucine | Valine, leucine and isoleucine degradation  Valine, leucine and isoleucine biosynthesis  Glucosinolate biosynthesis  Aminoacyl-tRNA biosynthesis  Biosynthesis of plant secondary metabolites  Biosynthesis of alkaloids derived from histidine and purine  Biosynthesis of secondary metabolites  2-Oxocarboxylic acid  Biosynthesis of amino acids  ABC transporters  Protein digestion and absorption  Mineral absorption  Shigellosis  Central carbon metabolism | Protein |
| 12 | Lysine | Lysine biosynthesis  Lysine degradation  D-Amino acid  Biotin metabolism  Tropane, piperidine and pyridine alkaloid biosynthesis  Aminoacyl – tRNA biosynthesis  Biosynthesis of secondary metabolites  Biosynthesis of alkaloids derived from ornithine, lysine and nicotinic acids  2-Oxocarboxylic acid  Biosynthesis of amino acids  ABC transporters  Protein digestion and absorption | Protein |
| 13 | N-Phenylacetylglycine | Phenyalanine  Glycine | Protein |
| 14 | Phenylacetate | Phenylalanine  Styrene degradation | Protein |
| 15 | Proline | Arginine and proline metabolism  Carbapenem biosynthesis  Prodigiosin biosynthesis  Novobiocin biosynthesis  Staurosporine biosynthesis  D-Amino acid metabolism  Aminoacyl-tRNA biosynthesis  Biosynthesis of secondary metabolites  Biosynthesis of amino acids  ABC transporters  Protein digestion and absorption  Mineral absorption  Central carbon metabolism in cancer | Protein |
| 16 | Tyrosine | Ubiquinone and other terpenoid-quinone biosynthesis  Monobactam biosynthesis  Tyrosine metabolism  Phenylalanine metabolism  Phenylalanine, tyrosine and tryptophan biosynthesis  Novobiocin biosynthesis  Cyanoamino acid metabolism  Methane metabolism  Thiamine metabolism  Phenylpropanoid biosynthesis  Isoquinoline alkaloid biosynthesis  Betalain biosynthesis  Glucosinolate biosynthesis  Aminoacyl-tRNA biosynthesis  Biosynthesis of various other secondary metabolites  Biosynthesis of various antibiotics  Biosynthesis of various plant secondary metabolites  Biosynthesis of vancomycin group antibiotics  Biosynthesis of enediyne antibiotics  Biosynthesis of plant secondary metabolites  Biosynthesis of alkaloids derived from shikimate pathway  Biosynthesis of secondary metabolites  2-Oxocarboxylic acid metabolism  Biosynthesis of amino acids  Biosynthesis of cofactors  Dopaminergic synapse  Melanogenesis  Prolactin signaling pathway  Protein digestion and absorption  Parkinson disease  Cocaine addiction  Amphetamine addiction  Alcoholism  Central carbon metabolism in cancer | Protein |
| 17 | Acetate | Glycolysis / Gluconeogenesis  Taurine and hypotaurine metabolism  Phosphonate and phosphinate metabolism  Glycosaminoglycan biosynthesis - heparan sulphate / heparin  Pyruvate metabolism  Glyoxylate and dicarboxylate metabolism  C5-Branched dibasic acid metabolism  Methane metabolism  Other carbon fixation pathways  Zeatin biosynthesis  Sulphur metabolism  Metabolic pathways  Biosynthesis of secondary metabolites  Microbial metabolism in diverse environments  Carbon metabolism  Degradation of aromatic compounds  Cholinergic synapse  Alcoholic liver disease  Carbohydrate digestion and absorption  Protein digestion and absorption | Carbohydrate |
| 18 | Butyrate | Butanoate metabolism  Metabolic pathways  Carbohydrate digestion and absorption  Protein digestion and absorption | Carbohydrate |
| 19 | Lactose | Galactose metabolism  ABC transporters  Phosphotransferase system (PTS)  Carbohydrate digestion and absorption | Carbohydrate |
| 20 | Maltose | Starch and sucrose metabolism  Metabolic pathways  Biosynthesis of secondary metabolites  ABC transporters  Bacterial chemotaxis  Phosphotransferase system (PTS)  Taste transduction  Carbohydrate digestion and absorption | Carbohydrate |
| 21 | Propionate | Propanoate metabolism  Ethylbenzene degradation  Nicotinate and nicotinamide metabolism  Metabolic pathways  Microbial metabolism in diverse environments  Degradation of aromatic compounds  Carbohydrate digestion and absorption  Protein digestion and absorption | Carbohydrate |
| 22 | 3-Phenylpropionate | Ethylbenzene degradation  Microbial metabolism in diverse environments  Degradation of aromatic compounds | Organic acids |
| 23 | Gentisate | Tyrosine metabolism  Benzoate degradation  Naphthalene degradation  Microbial metabolism in diverse environments | Organic acids |
| 24 | Maleate | Tyrosine metabolism  Butanoate metabolism  Nicotinate and nicotinamide metabolism  Microbial metabolism in diverse environments | Organic acids |
| 25 | Benzoate | Benzoate degradation  Dioxin degradation  Toluene degradation  Biosynthesis of various alkaloids  Biosynthesis of secondary metabolites  Degradation of aromatic compounds | Fatty Acid |
| 26 | Succinate | Citrate cycle (TCA cycle)  Oxidative phosphorylation  Alanine, aspartate and glutamate metabolism  Lysine degradation  Tyrosine metabolism  Phenylalanine metabolism  Chlorocyclohexane and chlorobenzene degradation  Pyruvate metabolism  Glyoxylate and dicarboxylate metabolism  Propanoate metabolism  Butanoate metabolism  Other carbon fixation pathways  Nicotinate and nicotinamide metabolism  Sulphur metabolism  Biosynthesis of plant secondary metabolites  Biosynthesis of phenylpropanoids  Biosynthesis of terpenoids and steroids  Biosynthesis of alkaloids derived from shikimate pathway  Biosynthesis of alkaloids derived from ornithine, lysine and nicotinic acid  Biosynthesis of alkaloids derived from histidine and purine  Biosynthesis of alkaloids derived from terpenoid and polyketide  Biosynthesis of plant hormones  Metabolic pathways  Biosynthesis of secondary metabolites  Microbial metabolism in diverse environments  Carbon metabolism  Degradation of aromatic compounds  Two-component system  cAMP signaling pathway  GABAergic synapse  Glucagon signaling pathway  Central carbon metabolism in cancer | Fatty acids |
| 27 | Pantothenate | beta-Alanine metabolism  Pantothenate and CoA biosynthesis  Metabolic pathways  Biosynthesis of secondary metabolites  Biosynthesis of cofactors  Vitamin digestion and absorption | Vitamin |
